# Supplementary figures and images for: Evidence of centromeric histone 3 chaperone involved in DNA damage repair pathway in budding yeast
Source: eLife. 2025 Dec 10;14:e104431. doi: 10.7554/eLife.104431 (PMC12695027; doi:10.7554/eLife.104431)

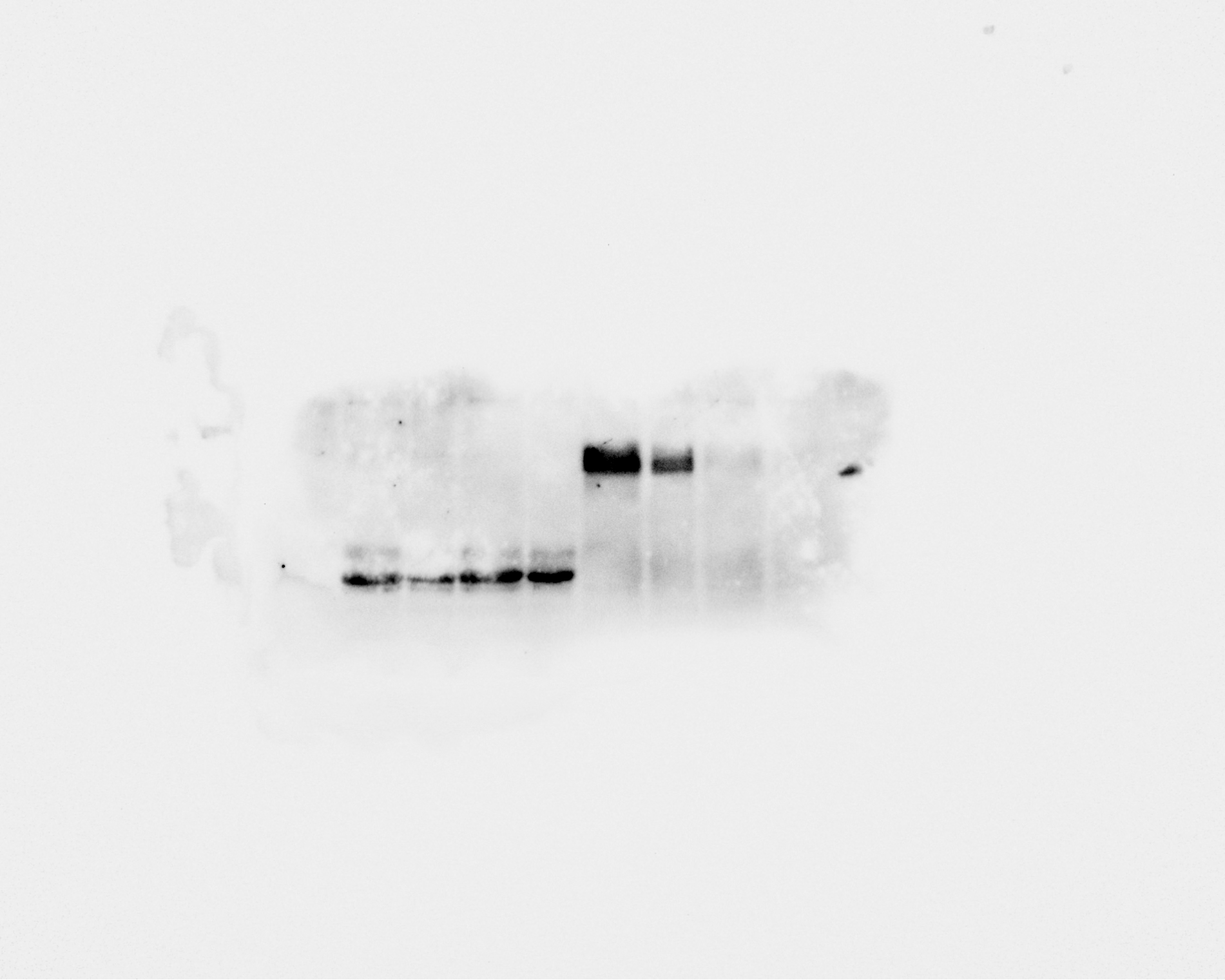

Supplement: Figure 1—figure supplement 2—source data 1. [file elife-104431-fig1-figsupp2-data1.zip › Figure 1- figure supplement 2/Figure 1-source data-Supp 2C Scm3.tif]

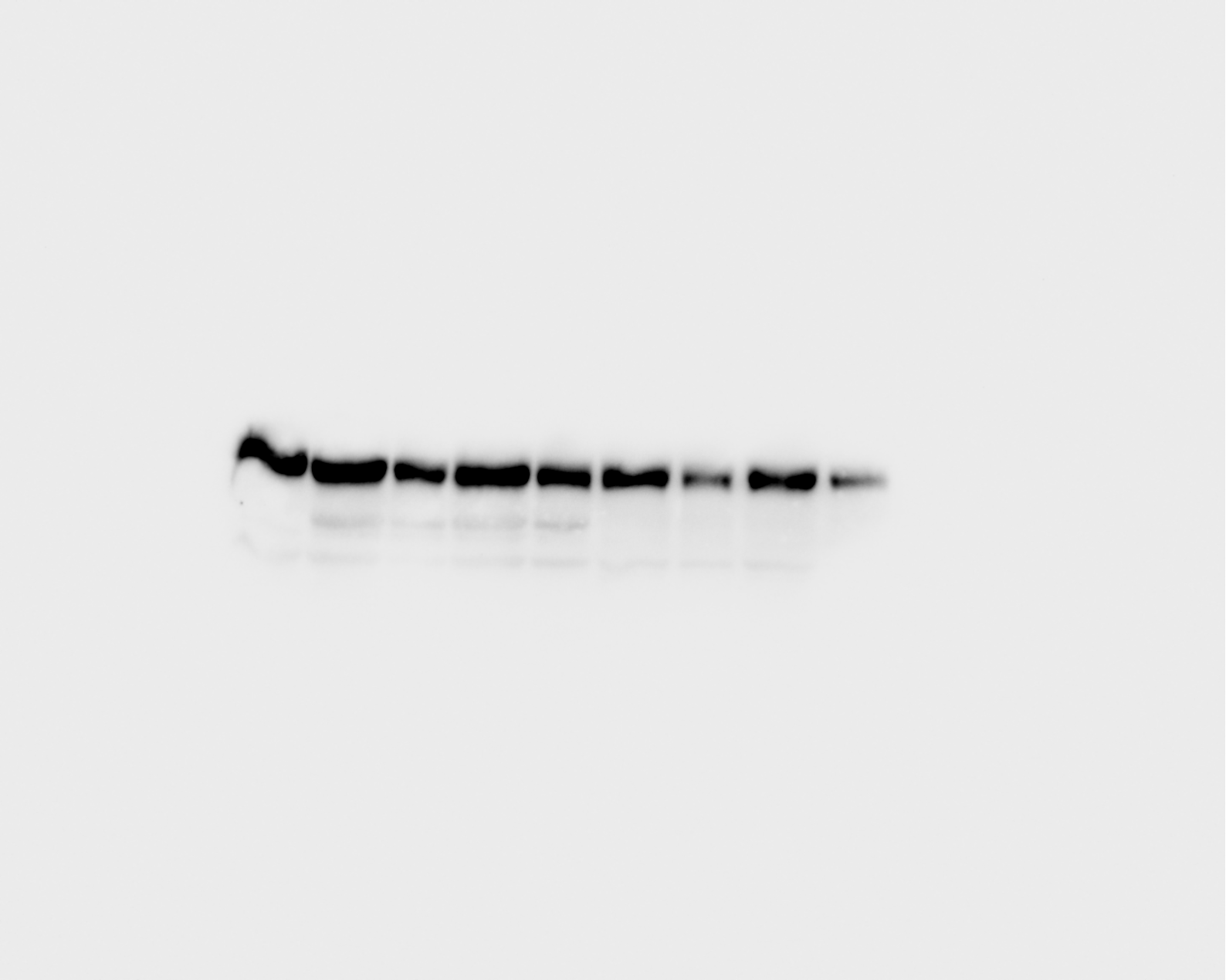

Supplement: Figure 1—figure supplement 2—source data 1. [file elife-104431-fig1-figsupp2-data1.zip › Figure 1- figure supplement 2/Figure 1-source data-Supp 2C Tub.tif]

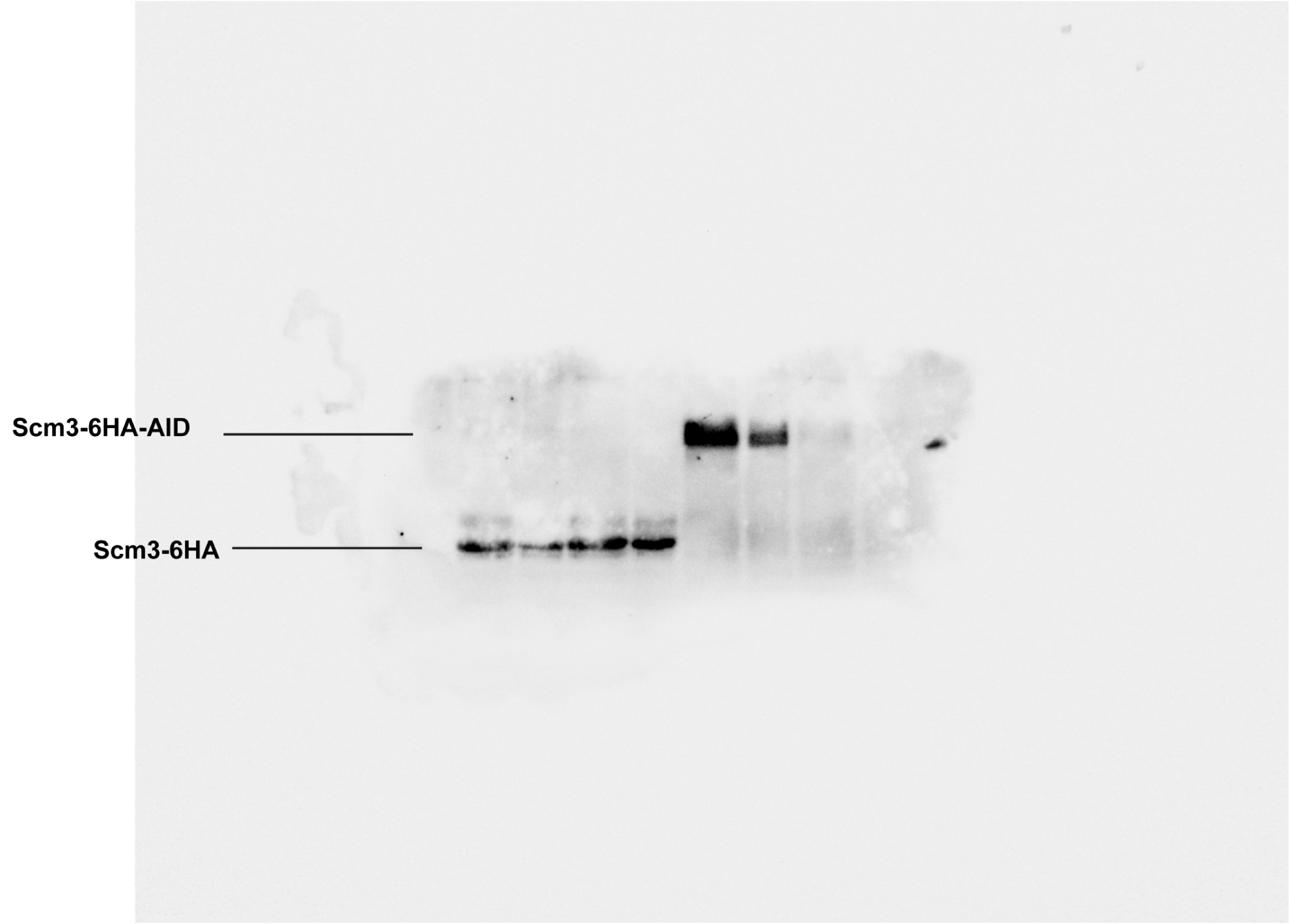

Supplement: Figure 1—figure supplement 2—source data 2. [file elife-104431-fig1-figsupp2-data2.zip › Figure 1- figure supplement 2/Figure 1-source data-Supp 2C Scm3.tif]

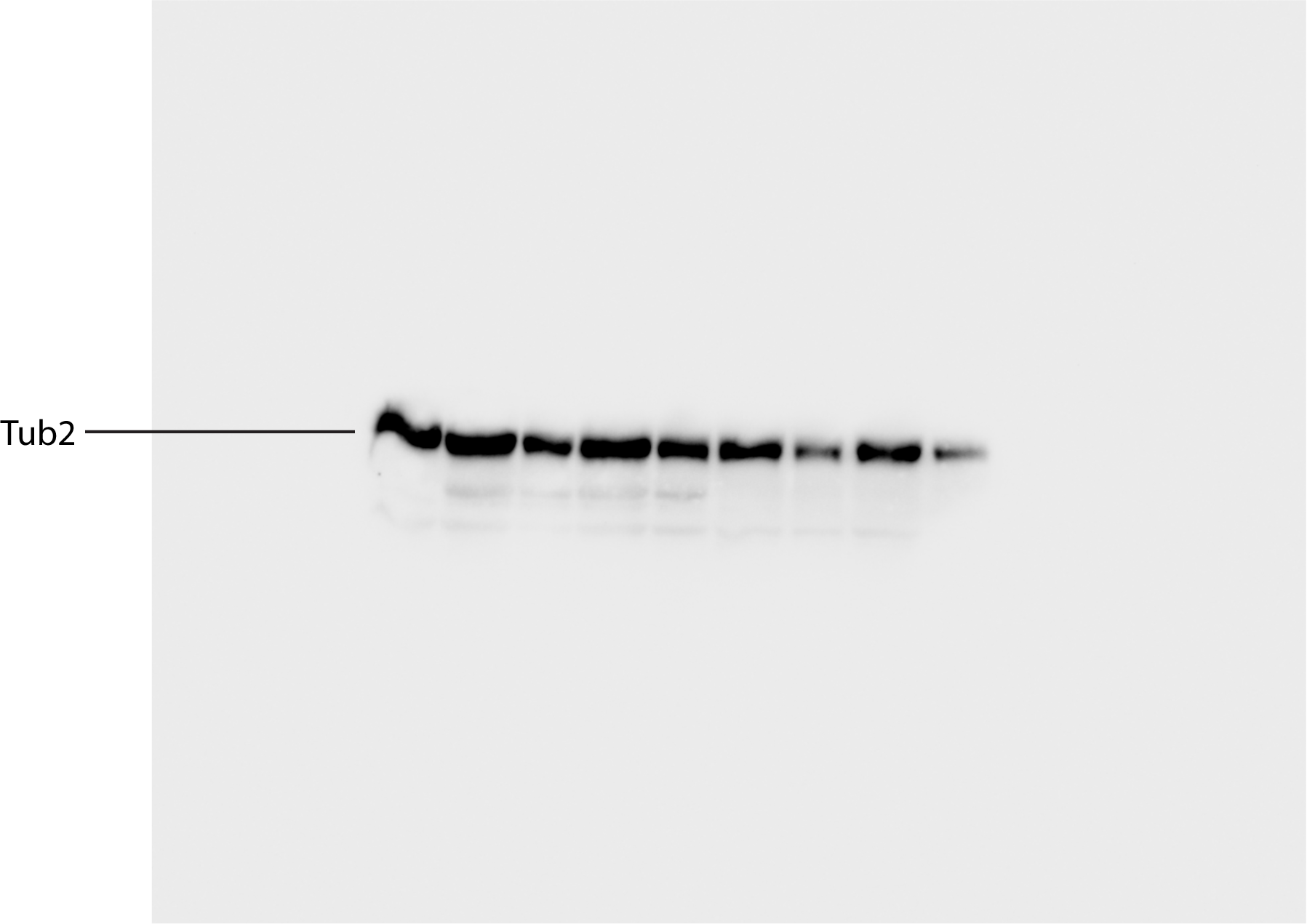

Supplement: Figure 1—figure supplement 2—source data 2. [file elife-104431-fig1-figsupp2-data2.zip › Figure 1- figure supplement 2/Figure 1-source data-Supp 2C Tub.tif]

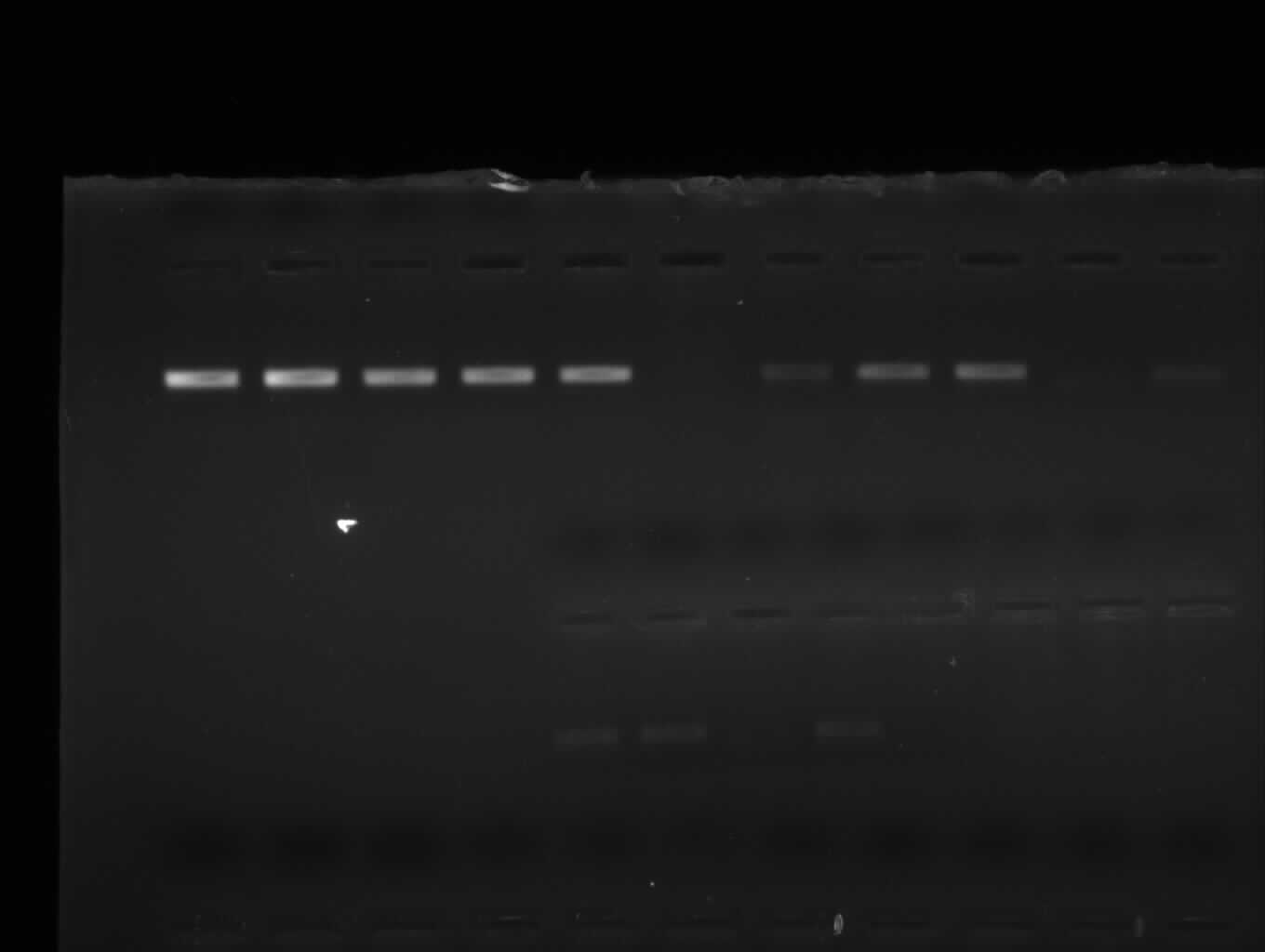

Supplement: Figure 5—source data 1. [file elife-104431-fig5-data1.zip › Figure 5/Figure 5-source data-5B Input.tif]

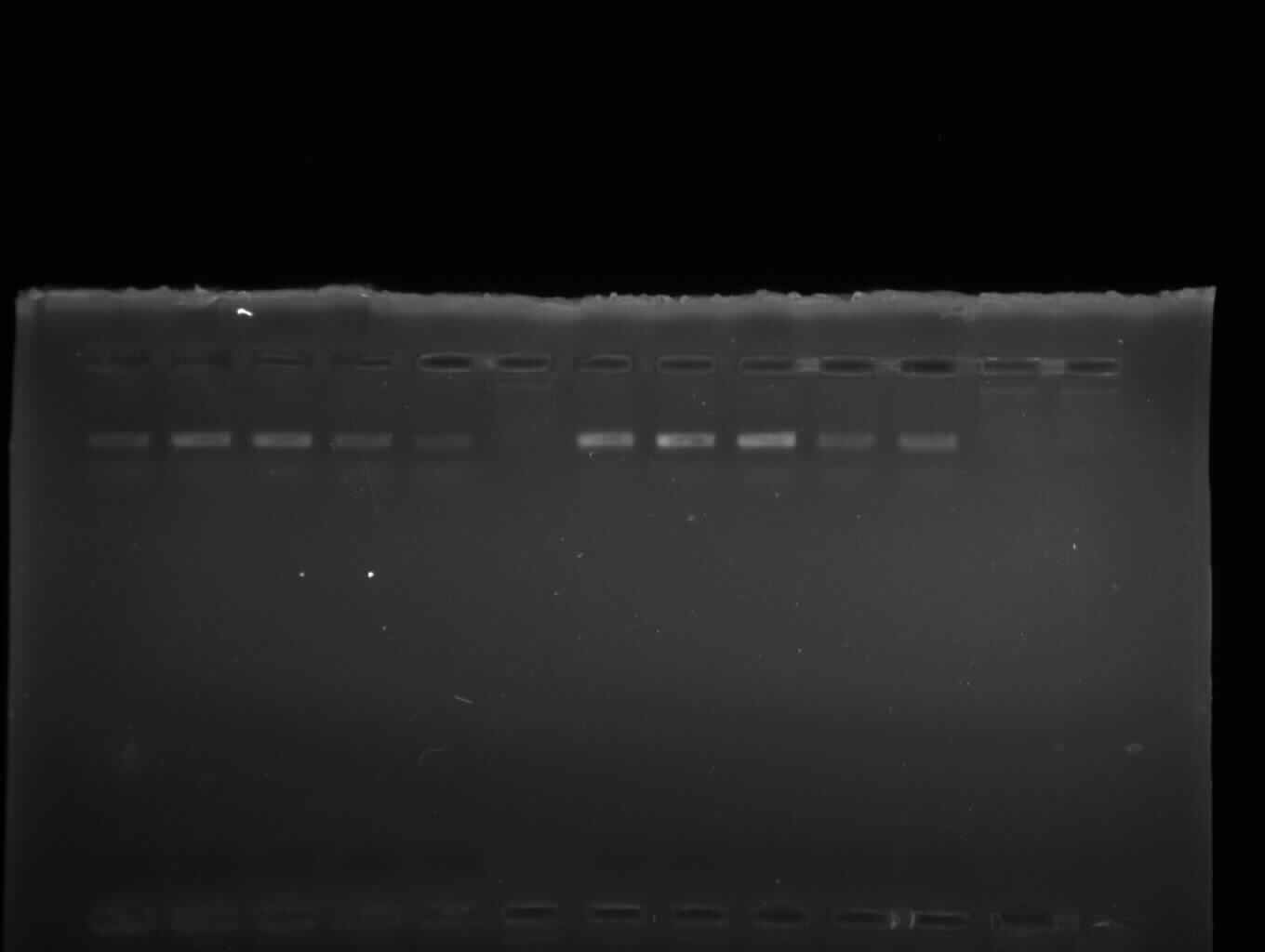

Supplement: Figure 5—source data 1. [file elife-104431-fig5-data1.zip › Figure 5/Figure 5-source data-5B Near DSB and CEN3.tif]

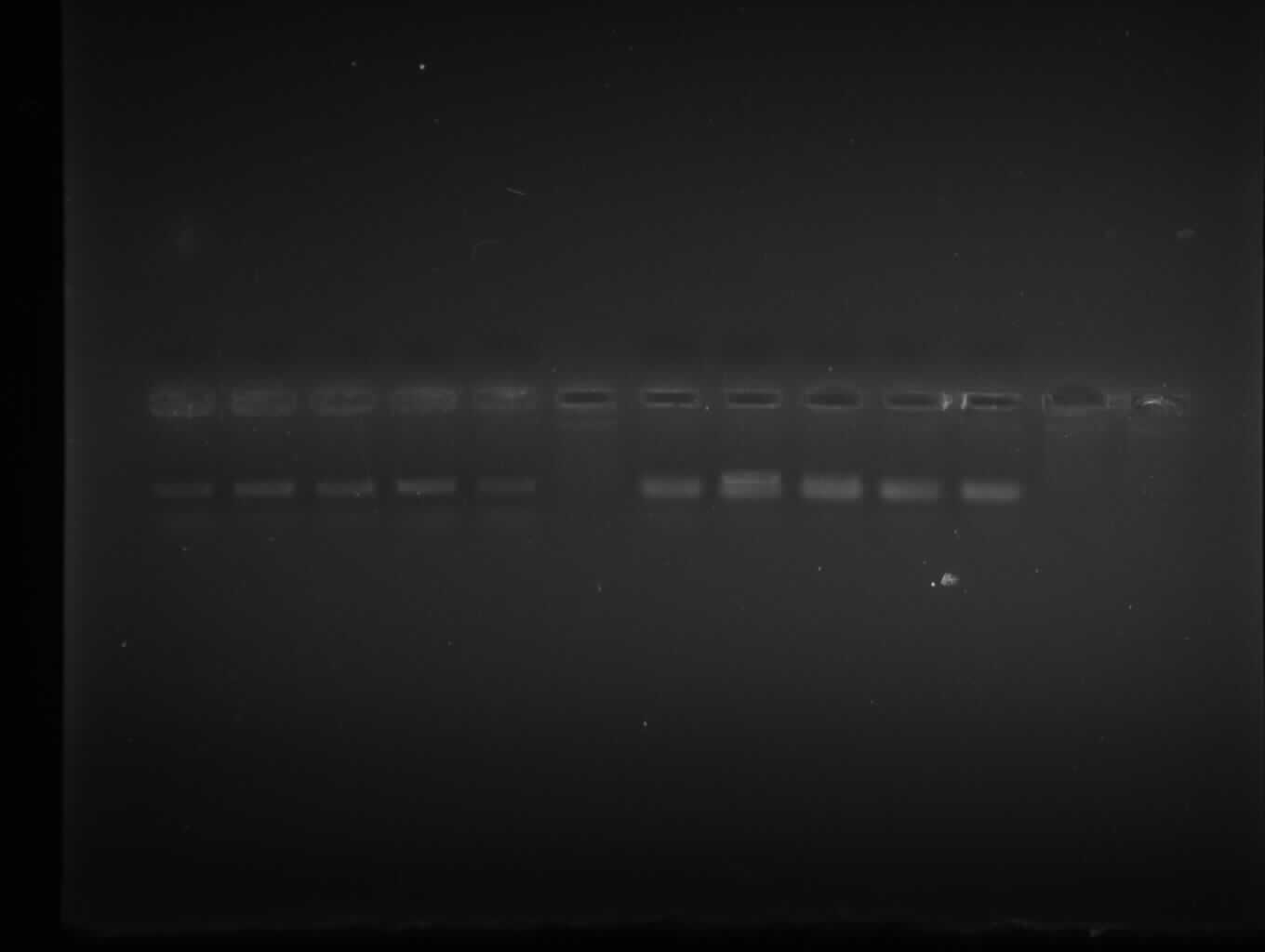

Supplement: Figure 5—source data 1. [file elife-104431-fig5-data1.zip › Figure 5/Figure 5-source data-5B TUB2.tif]

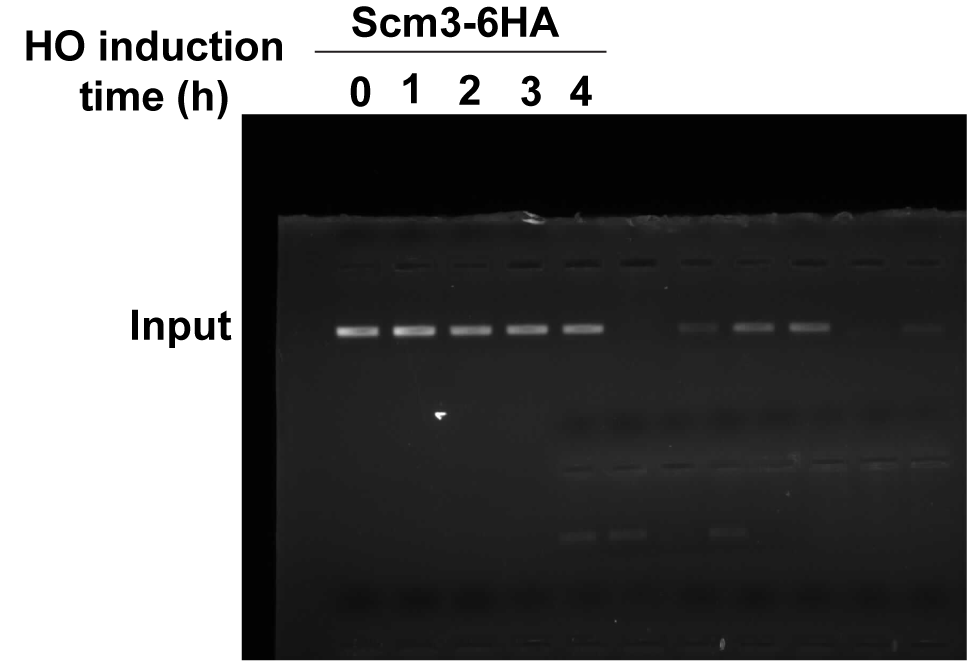

Supplement: Figure 5—source data 2. [file elife-104431-fig5-data2.zip › Figure 5 /Figure 5-source data-5B Input.tif]

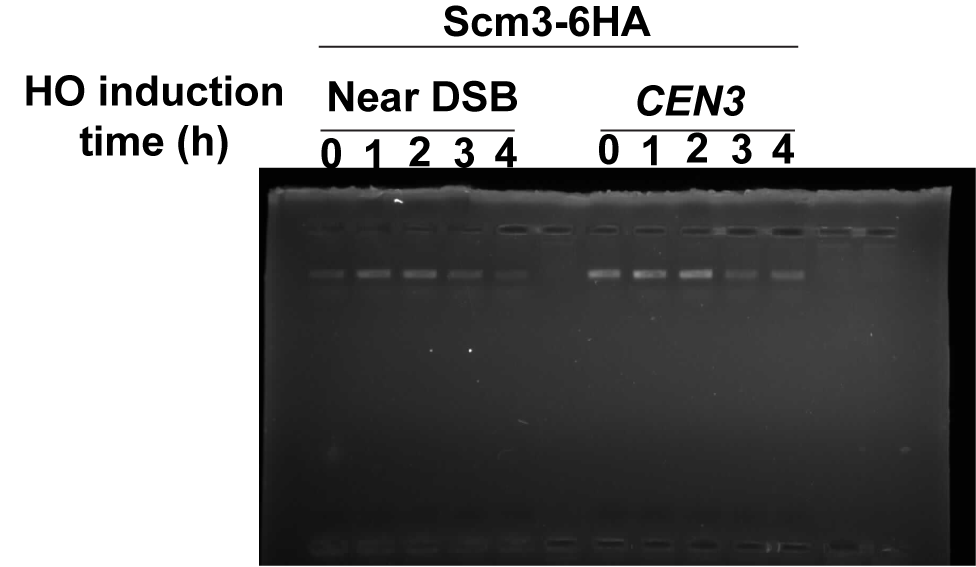

Supplement: Figure 5—source data 2. [file elife-104431-fig5-data2.zip › Figure 5 /Figure 5-source data-5B Near DSB and CEN3.tif]

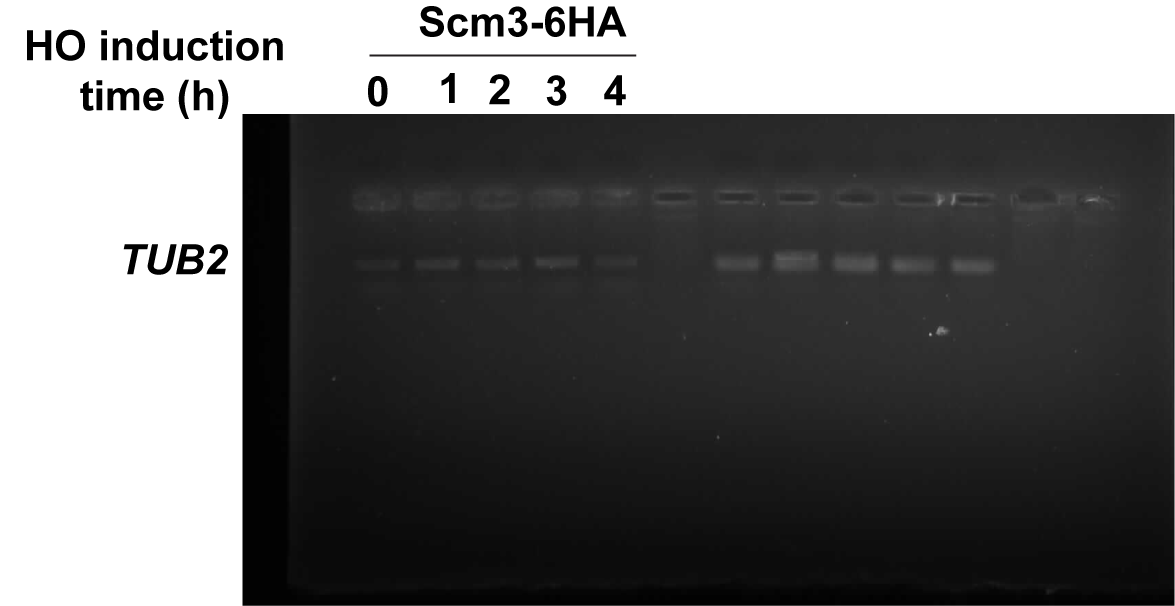

Supplement: Figure 5—source data 2. [file elife-104431-fig5-data2.zip › Figure 5 /Figure 5-source data-5B TUB2.tif]

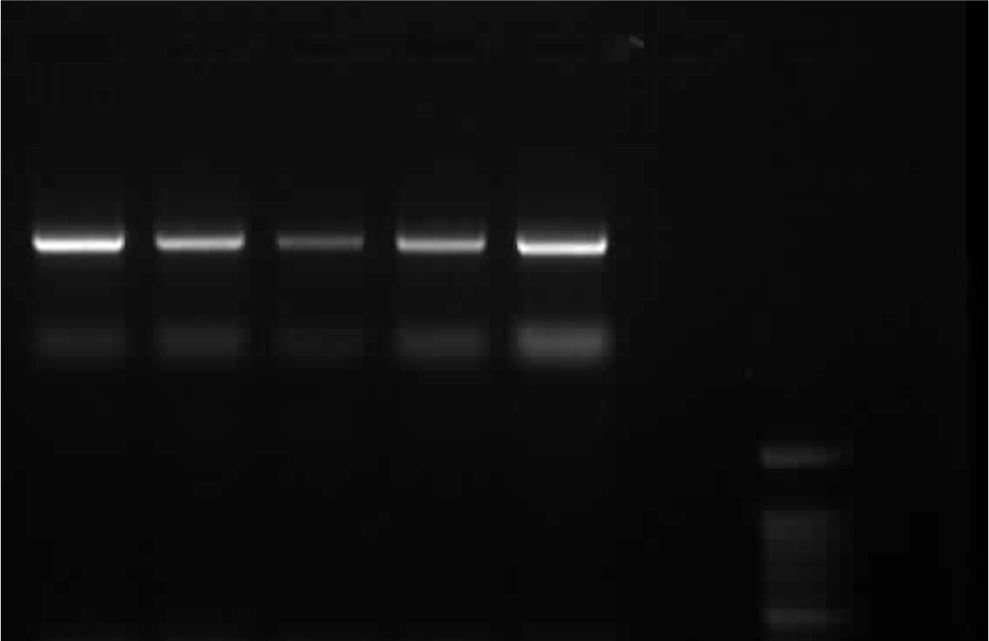

Supplement: Figure 5—figure supplement 1—source data 1. [file elife-104431-fig5-figsupp1-data1.zip › Figure 5- figure supplement 1/Figure 5-source data-Supp 1A DSB PCR.tif]

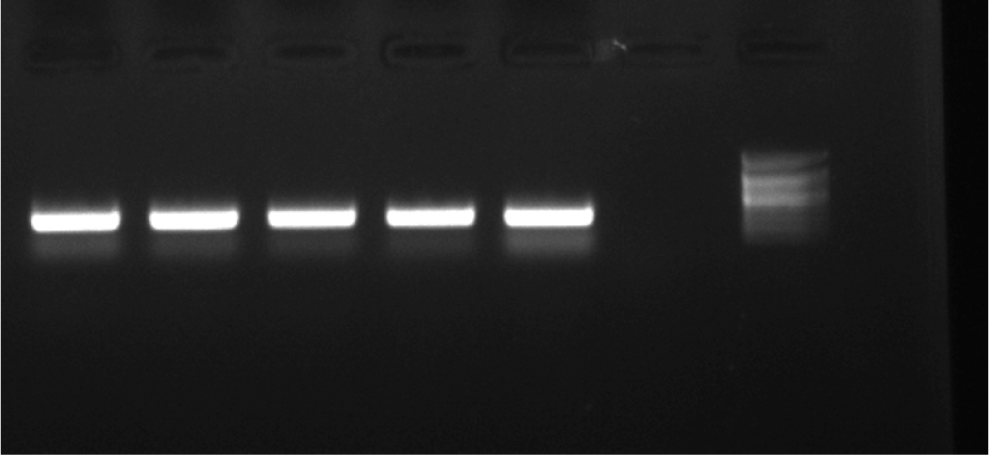

Supplement: Figure 5—figure supplement 1—source data 1. [file elife-104431-fig5-figsupp1-data1.zip › Figure 5- figure supplement 1/Figure 5-source data-Supp 1A TUB PCR.Tif]

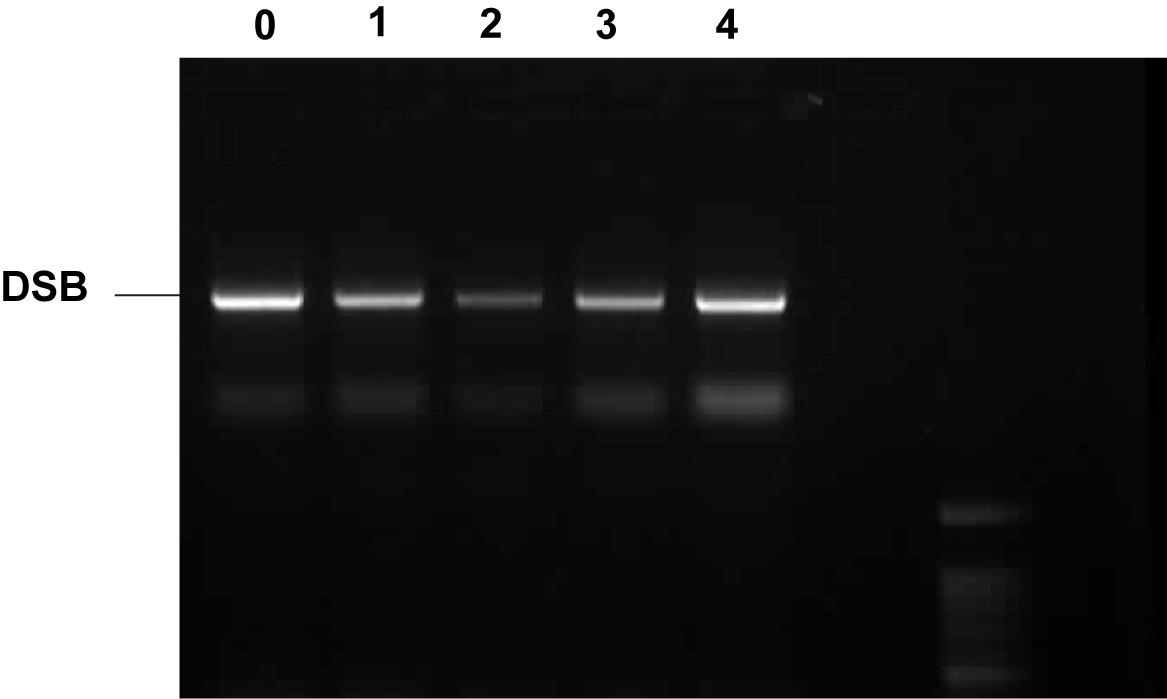

Supplement: Figure 5—figure supplement 1—source data 2. [file elife-104431-fig5-figsupp1-data2.zip › Figure 5- figure supplement 1 /Figure 5-source data-Supp 1A DSB PCR.tif]

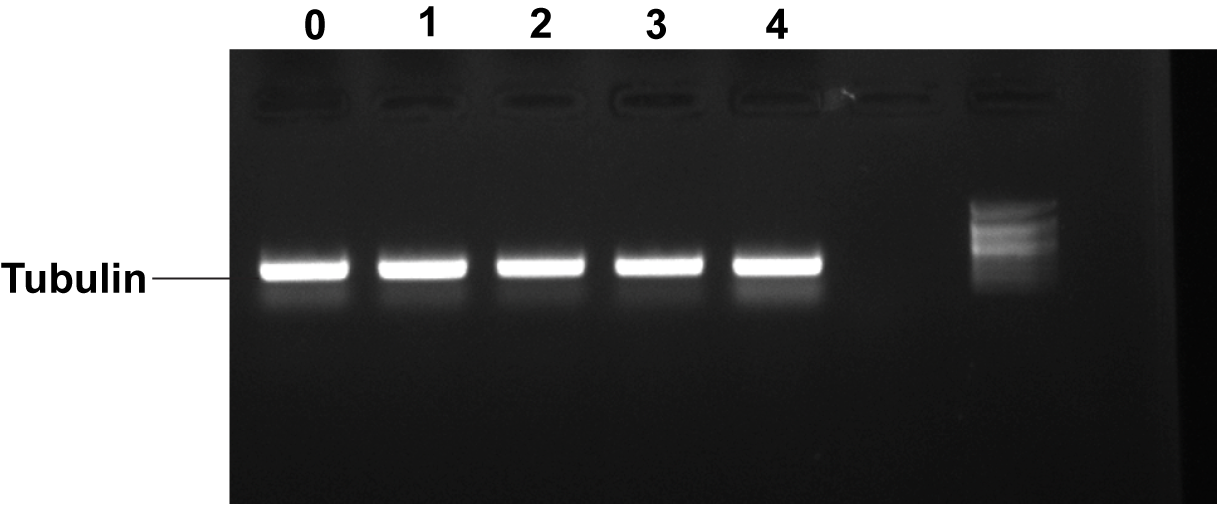

Supplement: Figure 5—figure supplement 1—source data 2. [file elife-104431-fig5-figsupp1-data2.zip › Figure 5- figure supplement 1 /Figure 5-source data-Supp 1A TUB PCR.Tif]

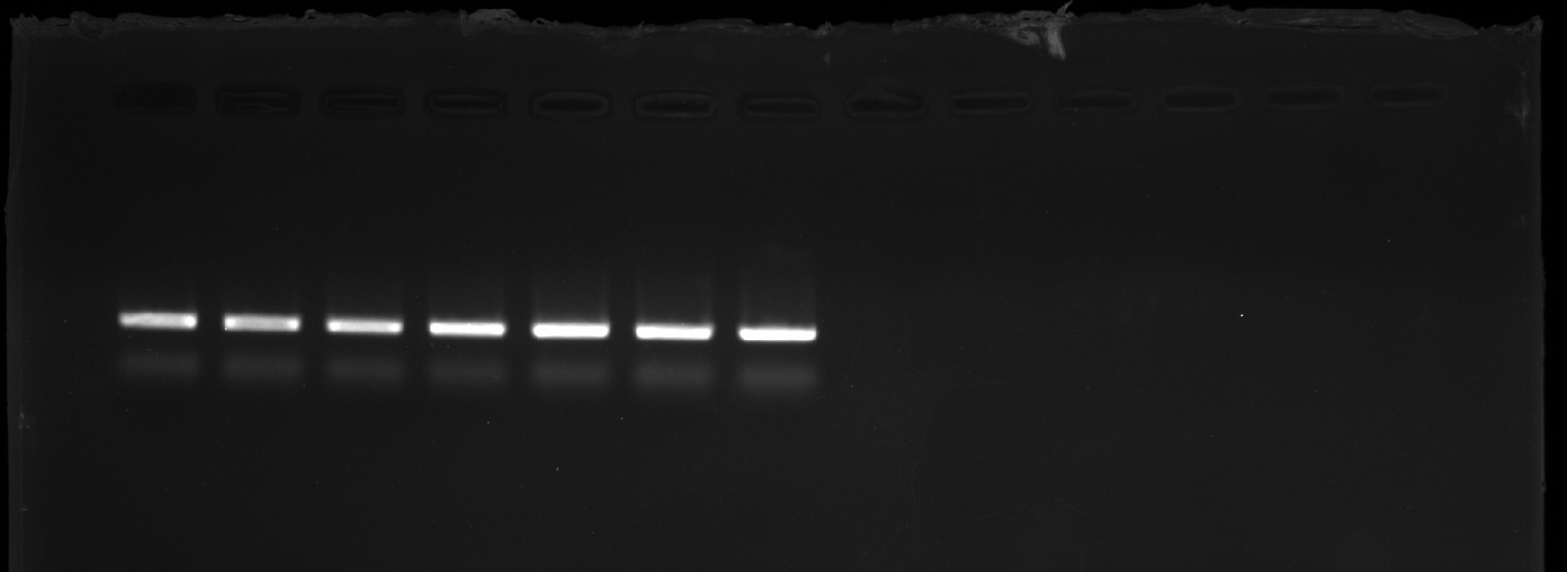

Supplement: Figure 5—figure supplement 2—source data 1. [file elife-104431-fig5-figsupp2-data1.zip › Figure 5- figure supplement 2/Figure 5-source data-Supp 2A SCM3 TUB2.tif]

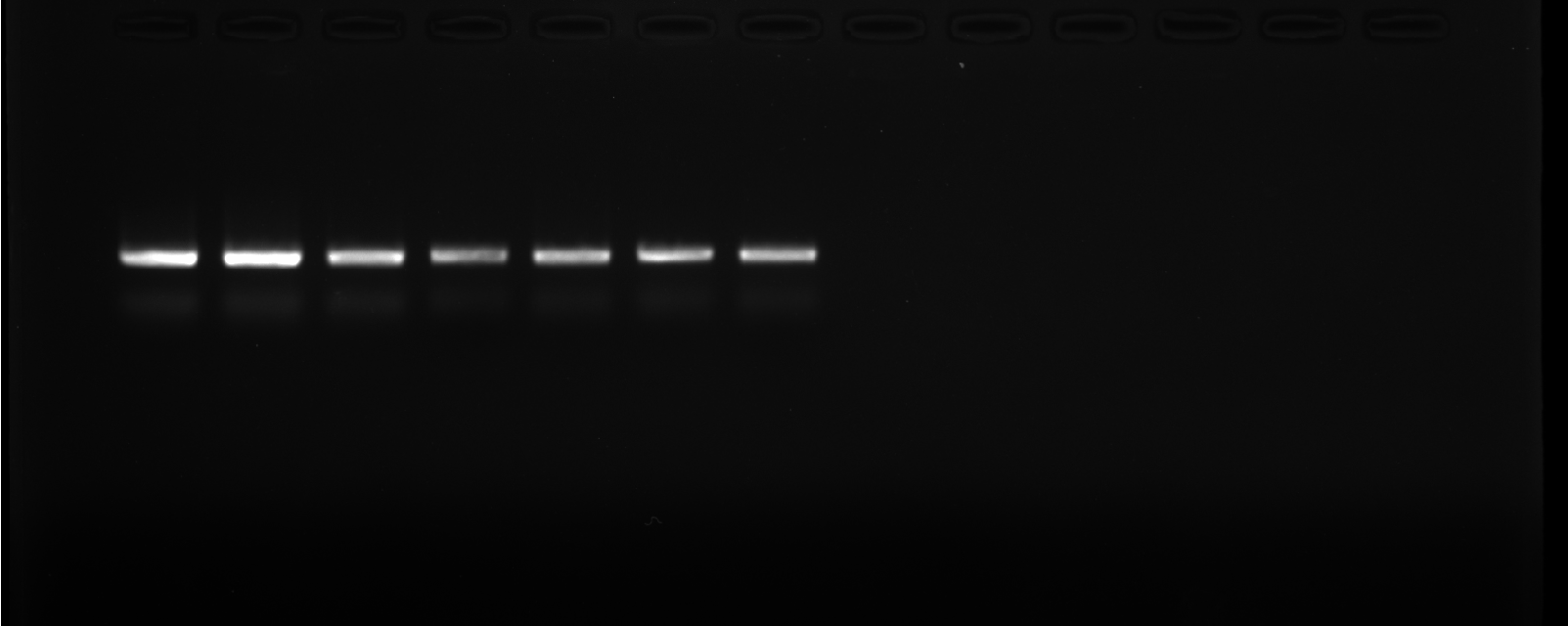

Supplement: Figure 5—figure supplement 2—source data 1. [file elife-104431-fig5-figsupp2-data1.zip › Figure 5- figure supplement 2/Figure 5-source data-Supp 2A SCM3-AID TUB.tif]

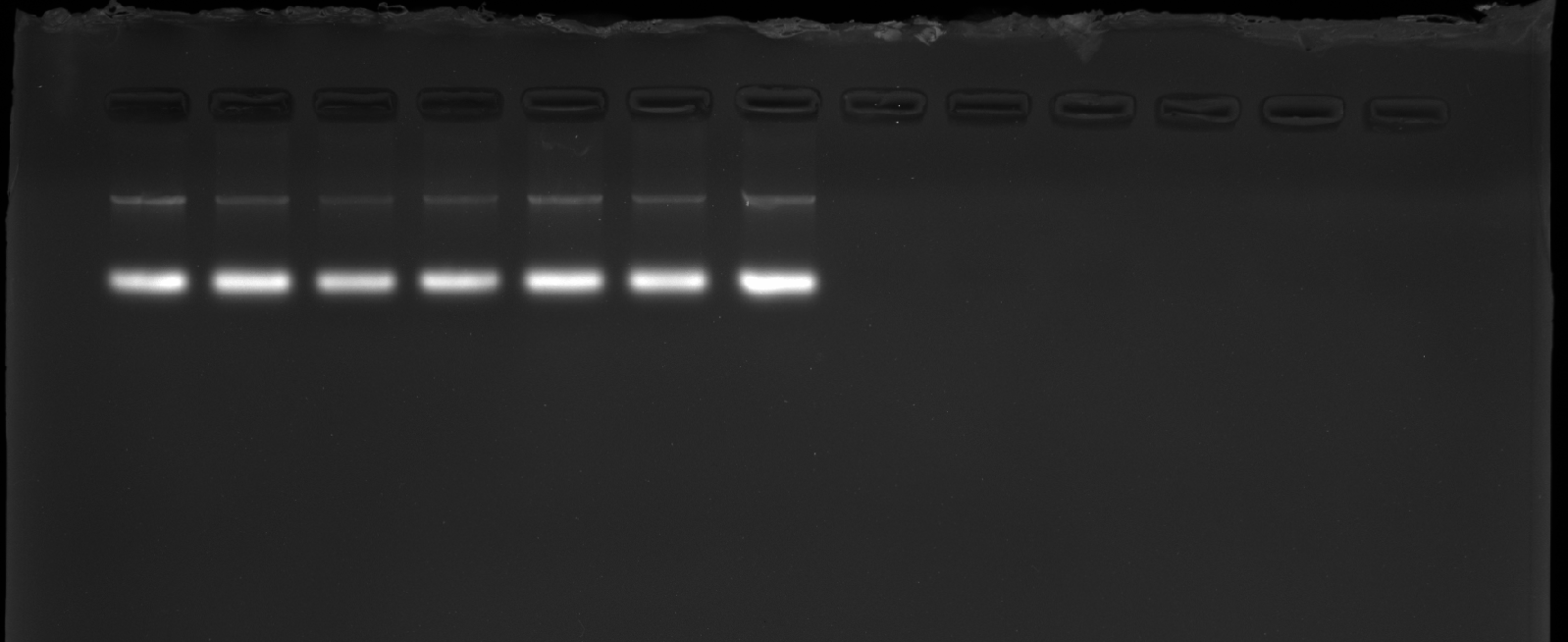

Supplement: Figure 5—figure supplement 2—source data 1. [file elife-104431-fig5-figsupp2-data1.zip › Figure 5- figure supplement 2/Figure 5-source data-Supp 2A SCM3 DSB.tif]

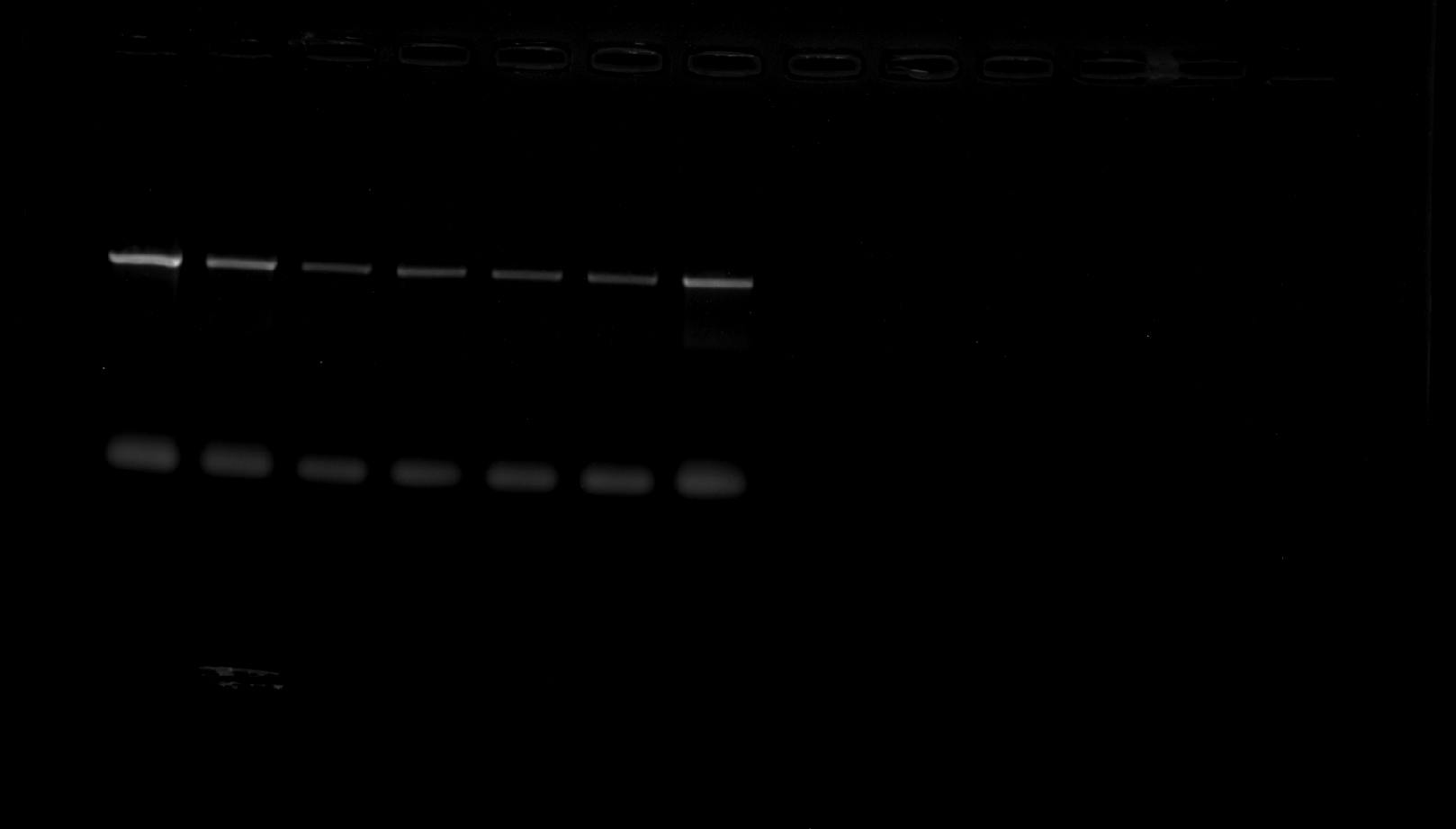

Supplement: Figure 5—figure supplement 2—source data 1. [file elife-104431-fig5-figsupp2-data1.zip › Figure 5- figure supplement 2/Figure 5-source data-Supp 2A SCM3-AID DSB.tif]

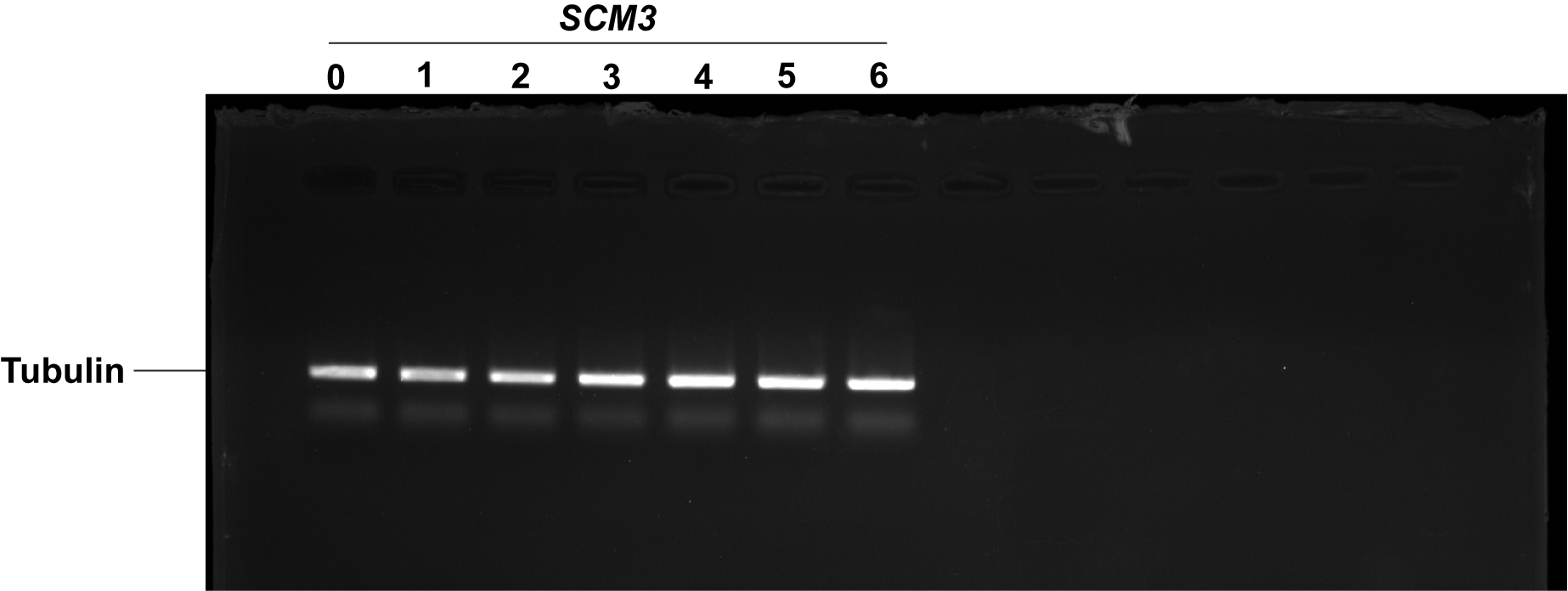

Supplement: Figure 5—figure supplement 2—source data 2. [file elife-104431-fig5-figsupp2-data2.zip › Figure 5- figure supplement 2 /Figure 5-source data-Supp 2A SCM3 TUB2.tif]

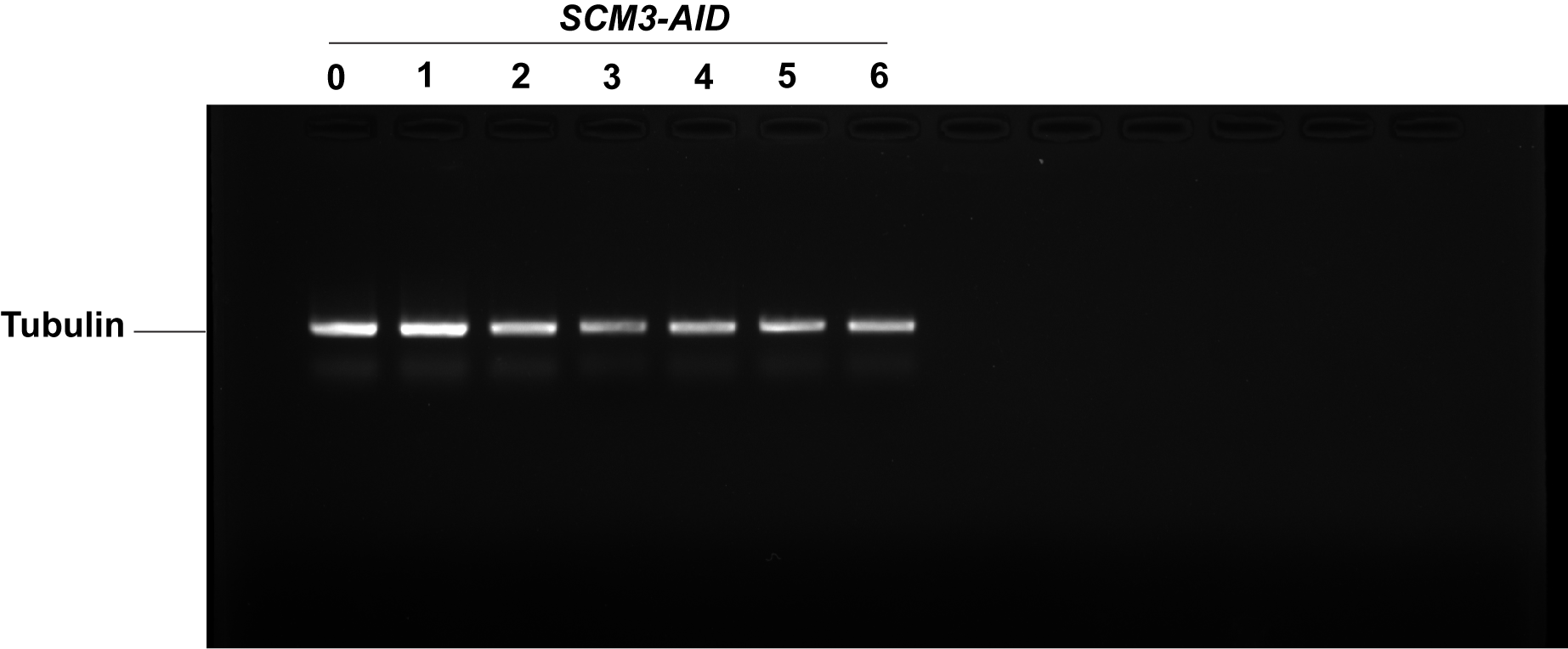

Supplement: Figure 5—figure supplement 2—source data 2. [file elife-104431-fig5-figsupp2-data2.zip › Figure 5- figure supplement 2 /Figure 5-source data-Supp 2A SCM3-AID TUB.tif]

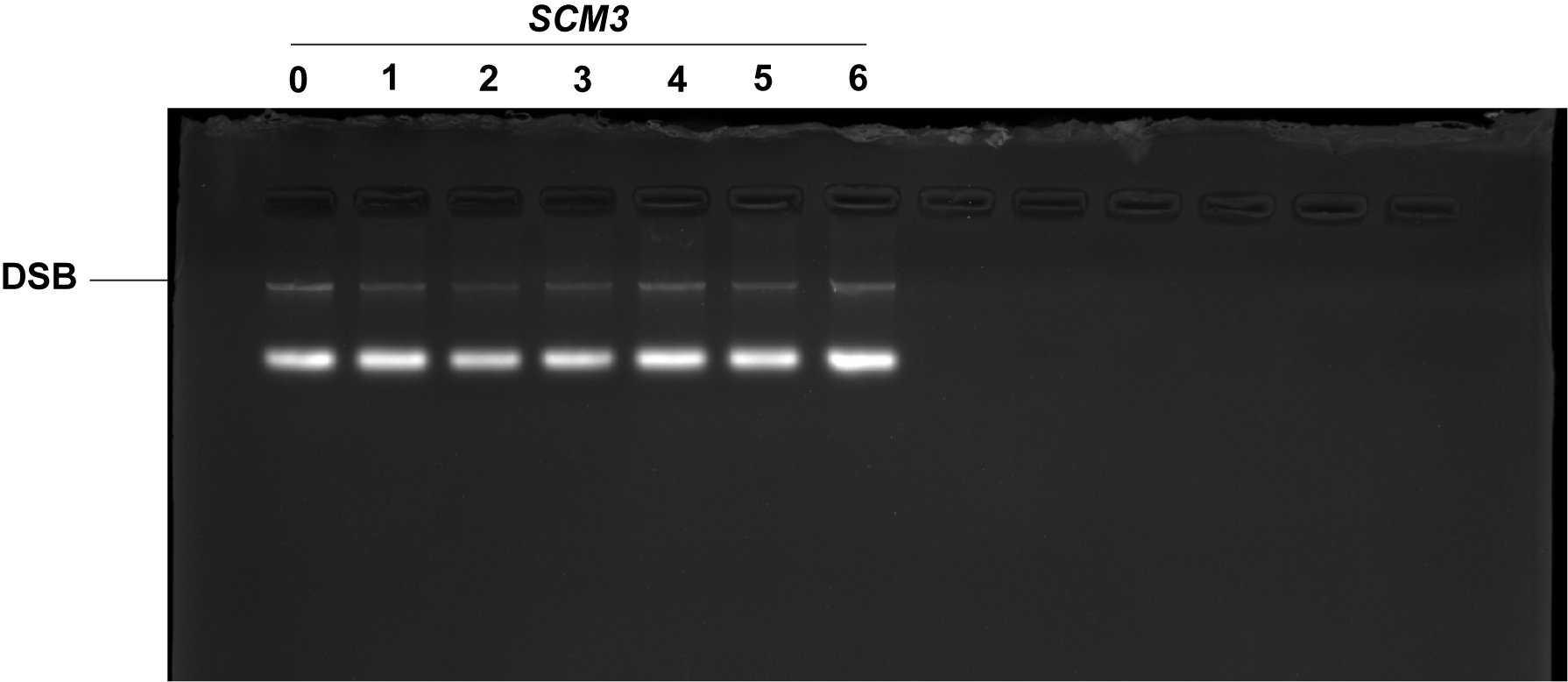

Supplement: Figure 5—figure supplement 2—source data 2. [file elife-104431-fig5-figsupp2-data2.zip › Figure 5- figure supplement 2 /Figure 5-source data-Supp 2A SCM3 DSB.tif]

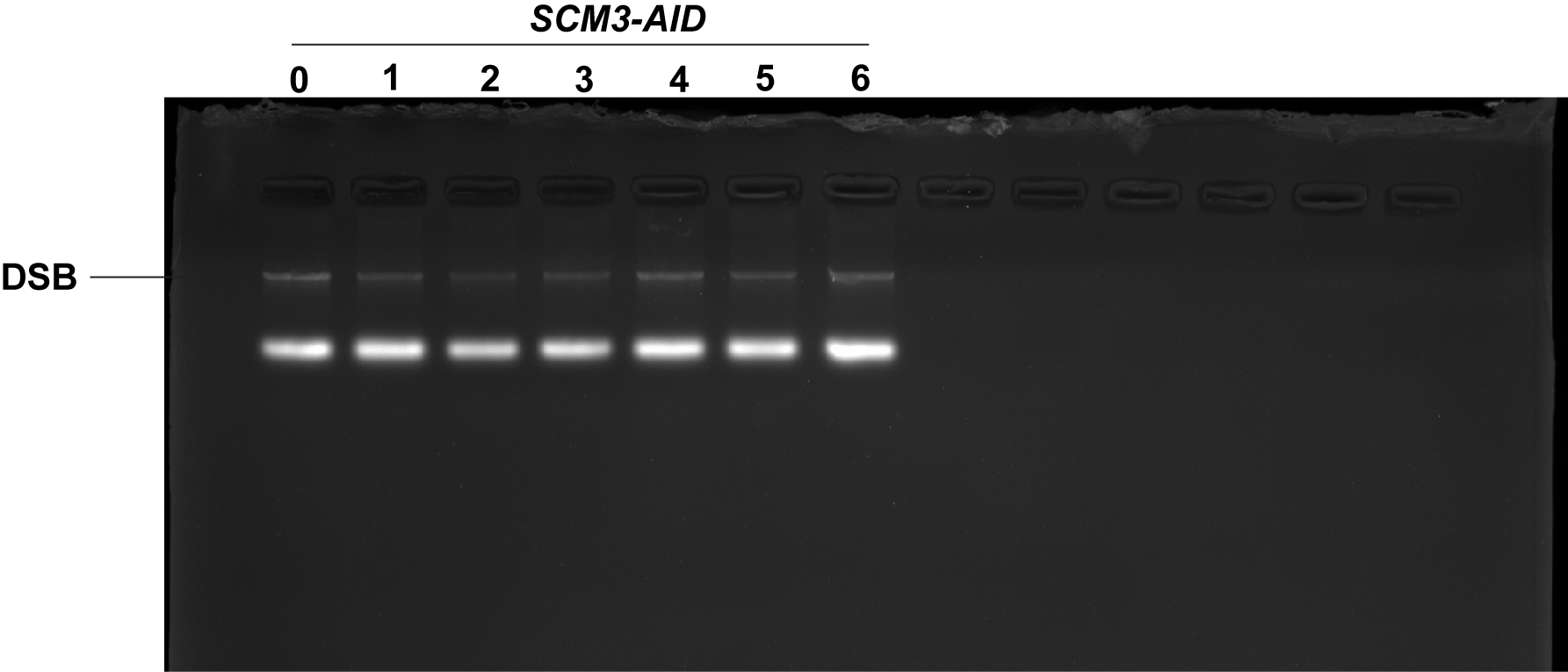

Supplement: Figure 5—figure supplement 2—source data 2. [file elife-104431-fig5-figsupp2-data2.zip › Figure 5- figure supplement 2 /Figure 5-source data-Supp 2A SCM3-AID DSB.tif]

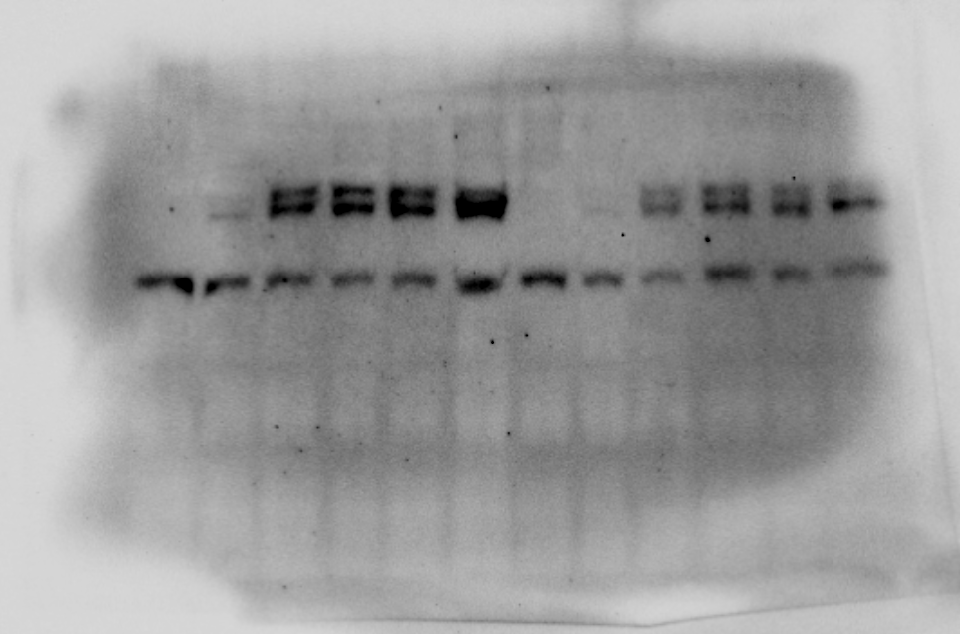

Supplement: Figure 6—source data 1. [file elife-104431-fig6-data1.zip › Figure 6/Figure 6-source data-6E.tif]

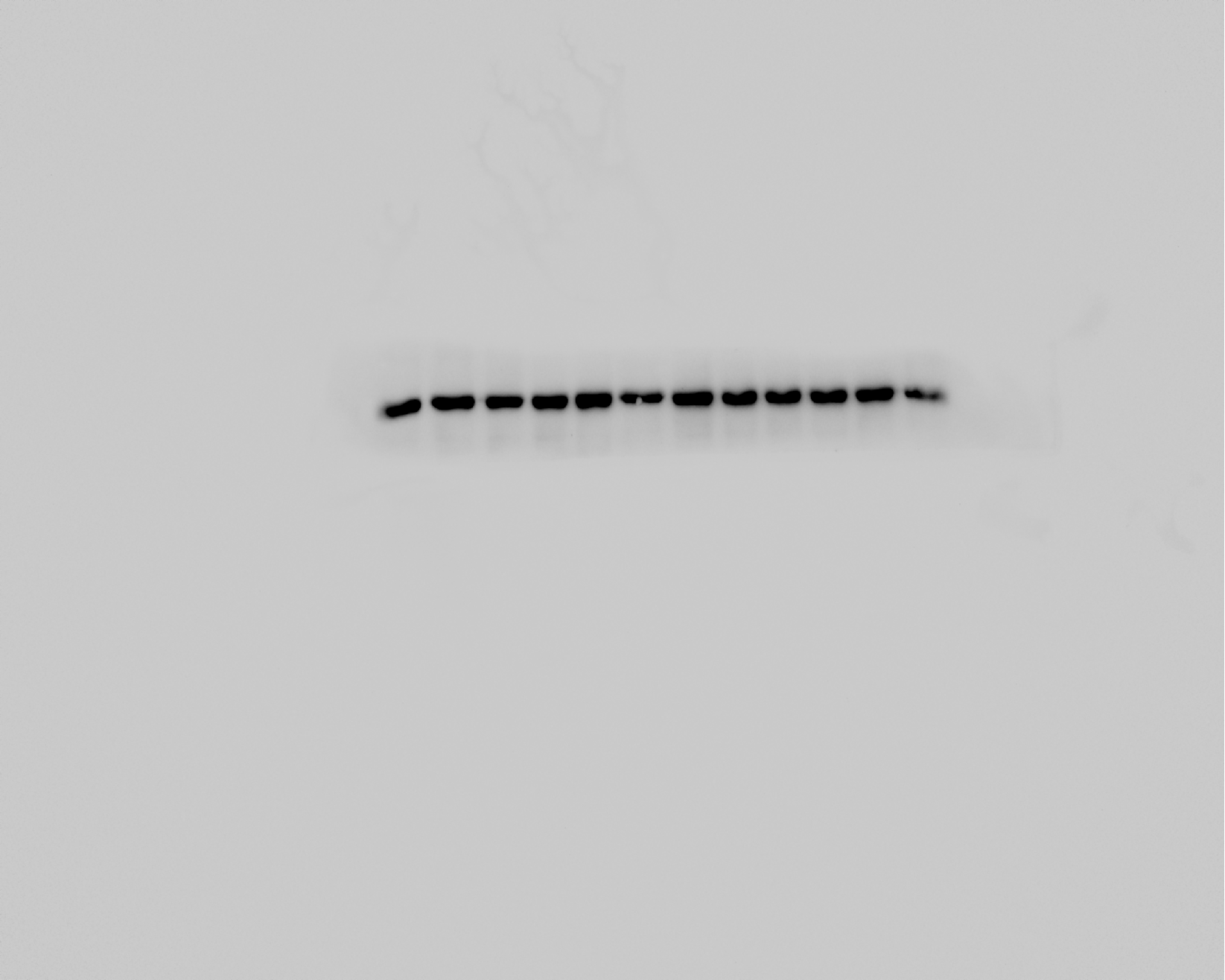

Supplement: Figure 6—source data 1. [file elife-104431-fig6-data1.zip › Figure 6/Figure 6-source data-6B_Tub.tif]

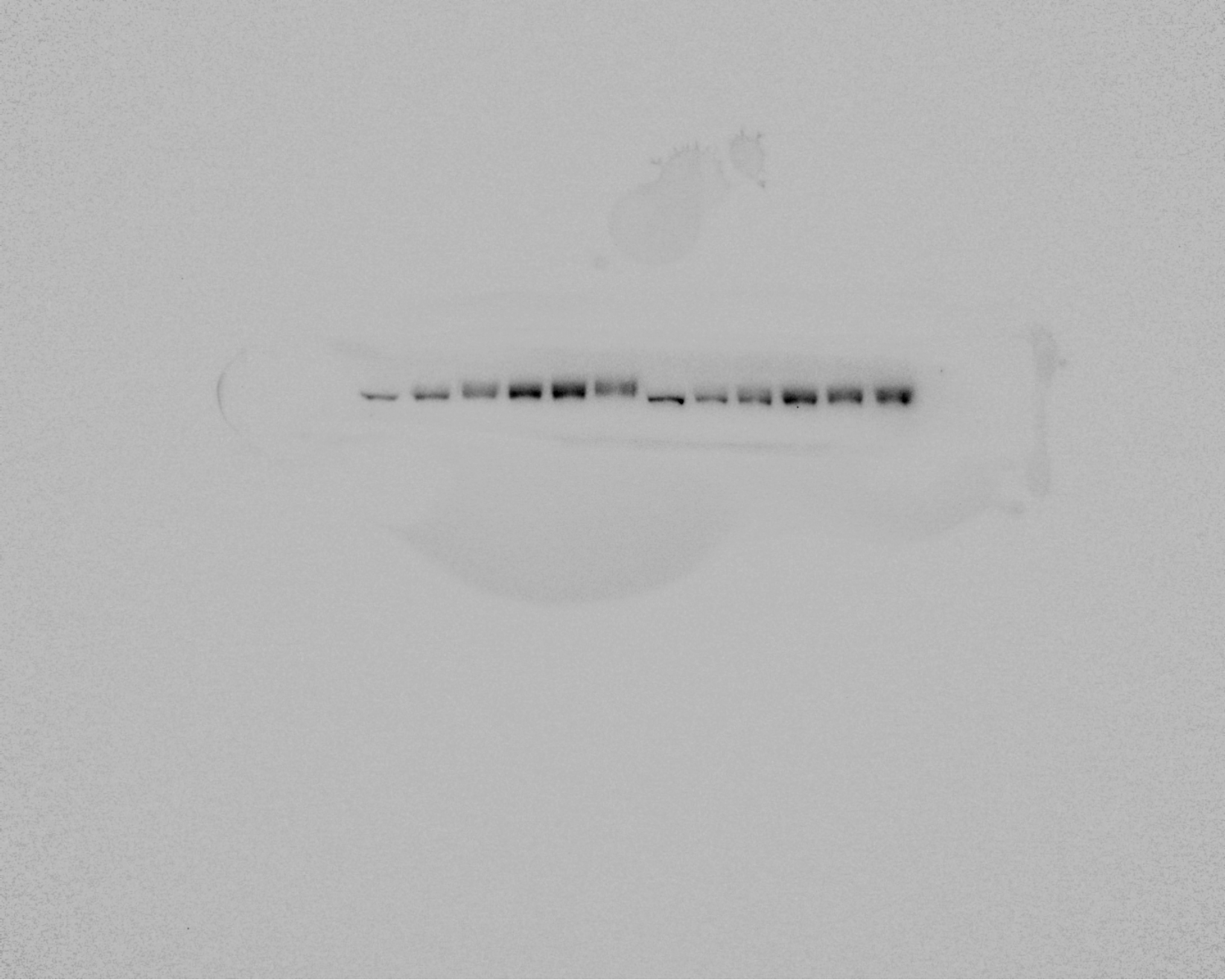

Supplement: Figure 6—source data 1. [file elife-104431-fig6-data1.zip › Figure 6/Figure 6-source data-6B_Rad53.tif]

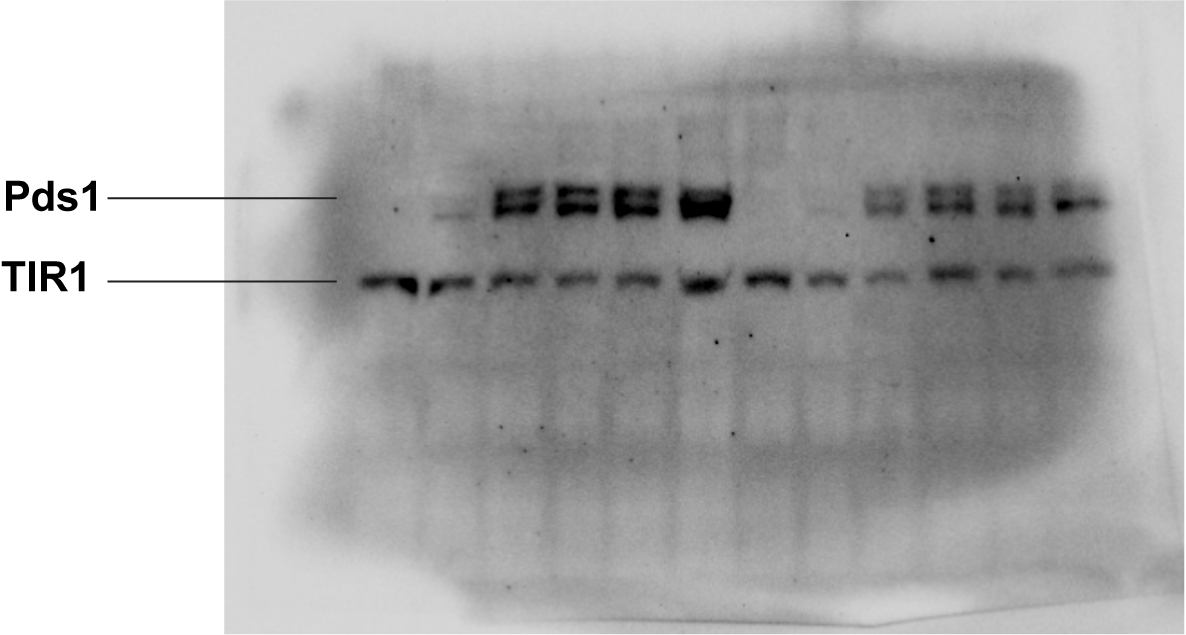

Supplement: Figure 6—source data 2. [file elife-104431-fig6-data2.zip › Figure 6/Figure 6-source data-6E.tif]

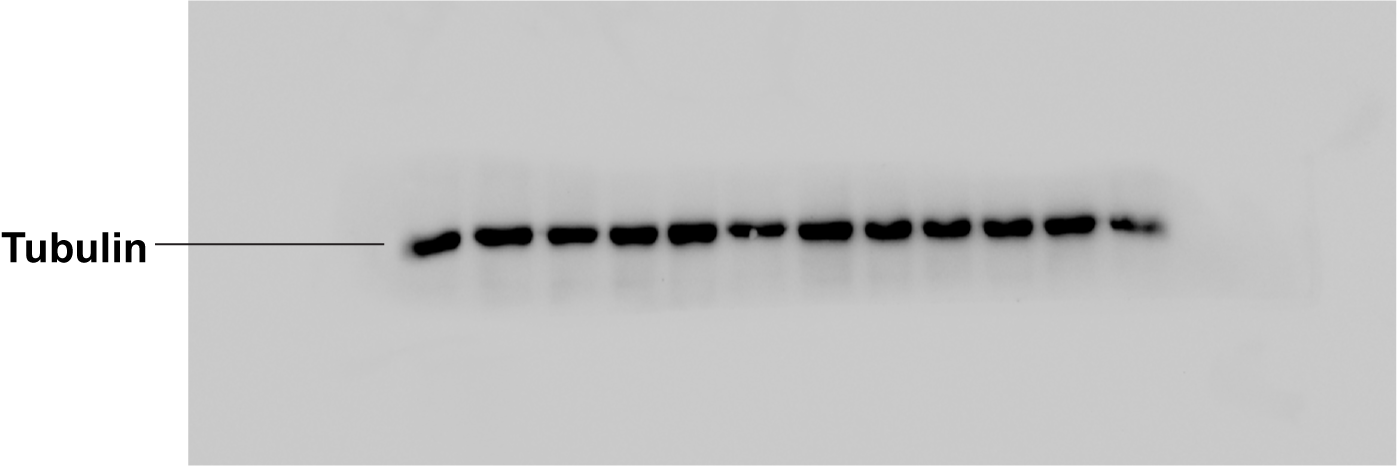

Supplement: Figure 6—source data 2. [file elife-104431-fig6-data2.zip › Figure 6/Figure 6-source data-6B_Tub.tif]

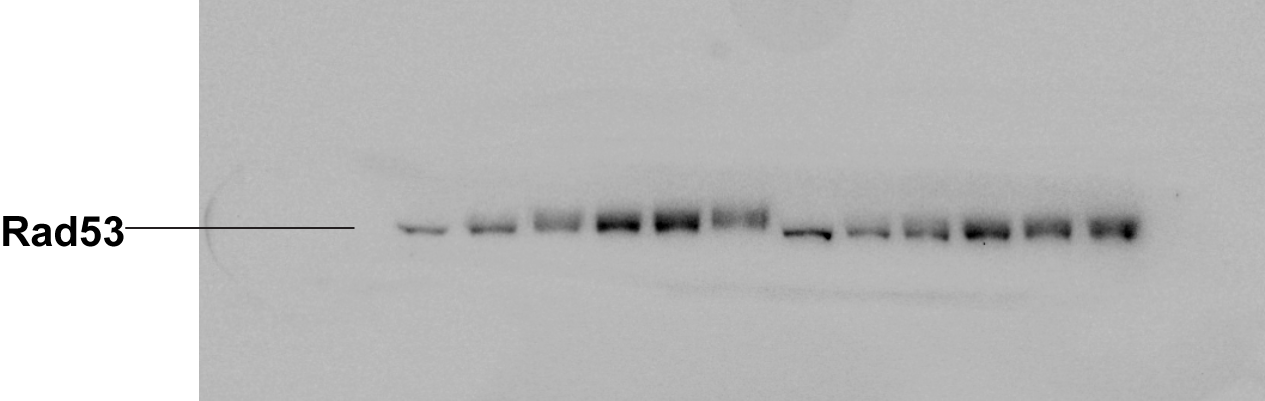

Supplement: Figure 6—source data 2. [file elife-104431-fig6-data2.zip › Figure 6/Figure 6-source data-6B_Rad53.tif]

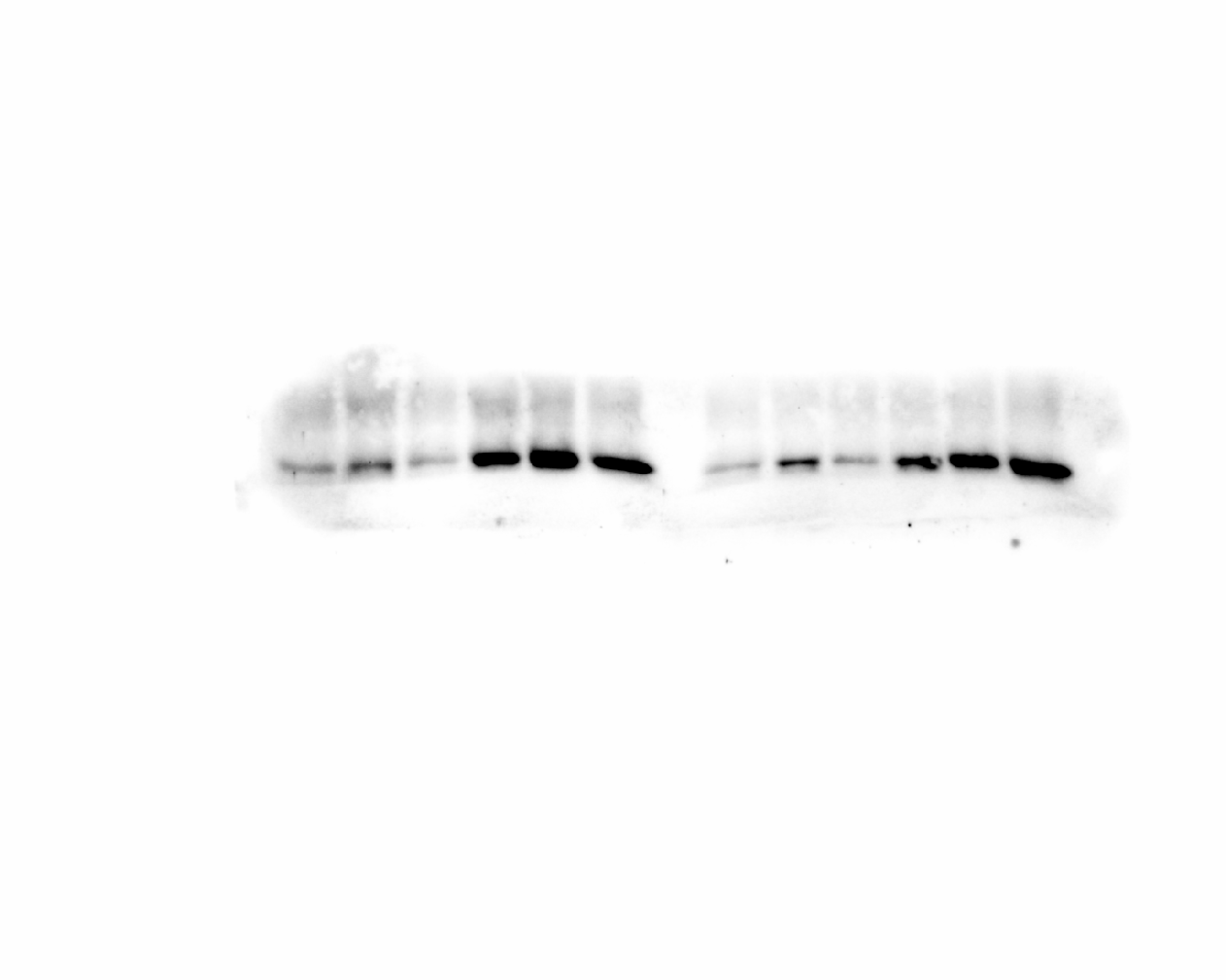

Supplement: Figure 6—figure supplement 1—source data 1. [file elife-104431-fig6-figsupp1-data1.zip › Figure 6- figure supplement 1 /Figure 6-source data-Supp 6F H2A.tif]

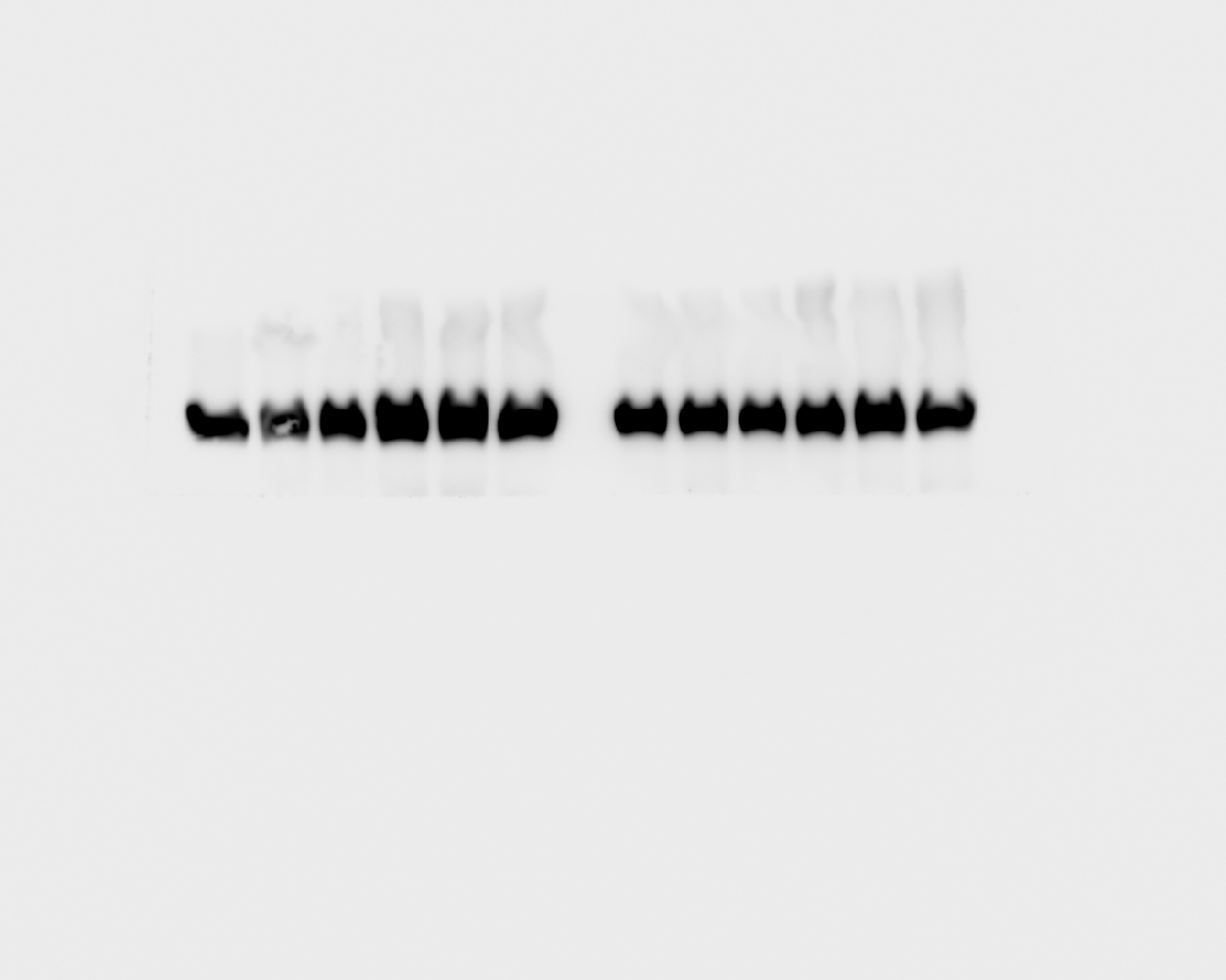

Supplement: Figure 6—figure supplement 1—source data 1. [file elife-104431-fig6-figsupp1-data1.zip › Figure 6- figure supplement 1 /Figure 6-source data-Supp 6F Tub.tif]

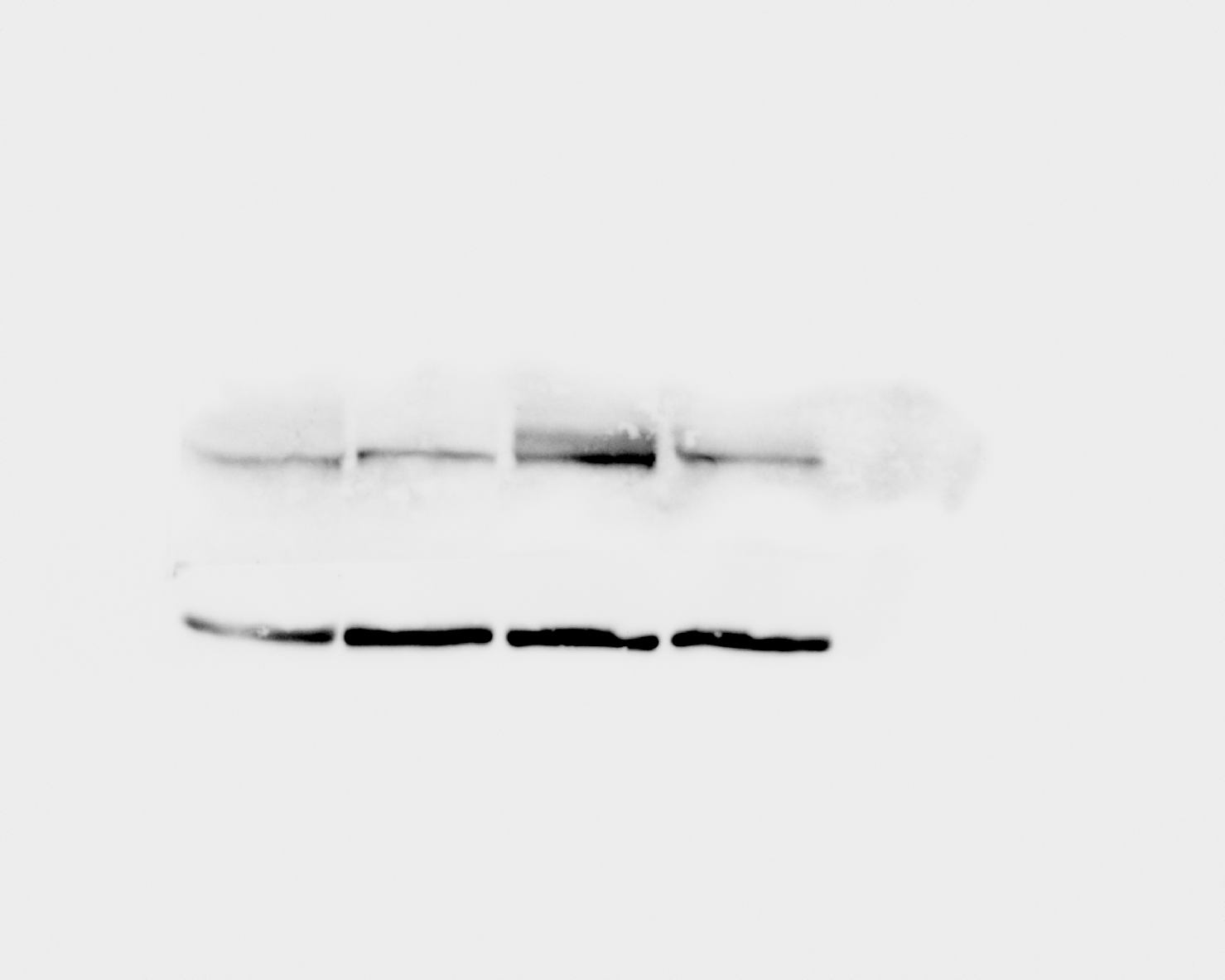

Supplement: Figure 6—figure supplement 1—source data 1. [file elife-104431-fig6-figsupp1-data1.zip › Figure 6- figure supplement 1 /Figure 6-source data-Supp 6B.tif]

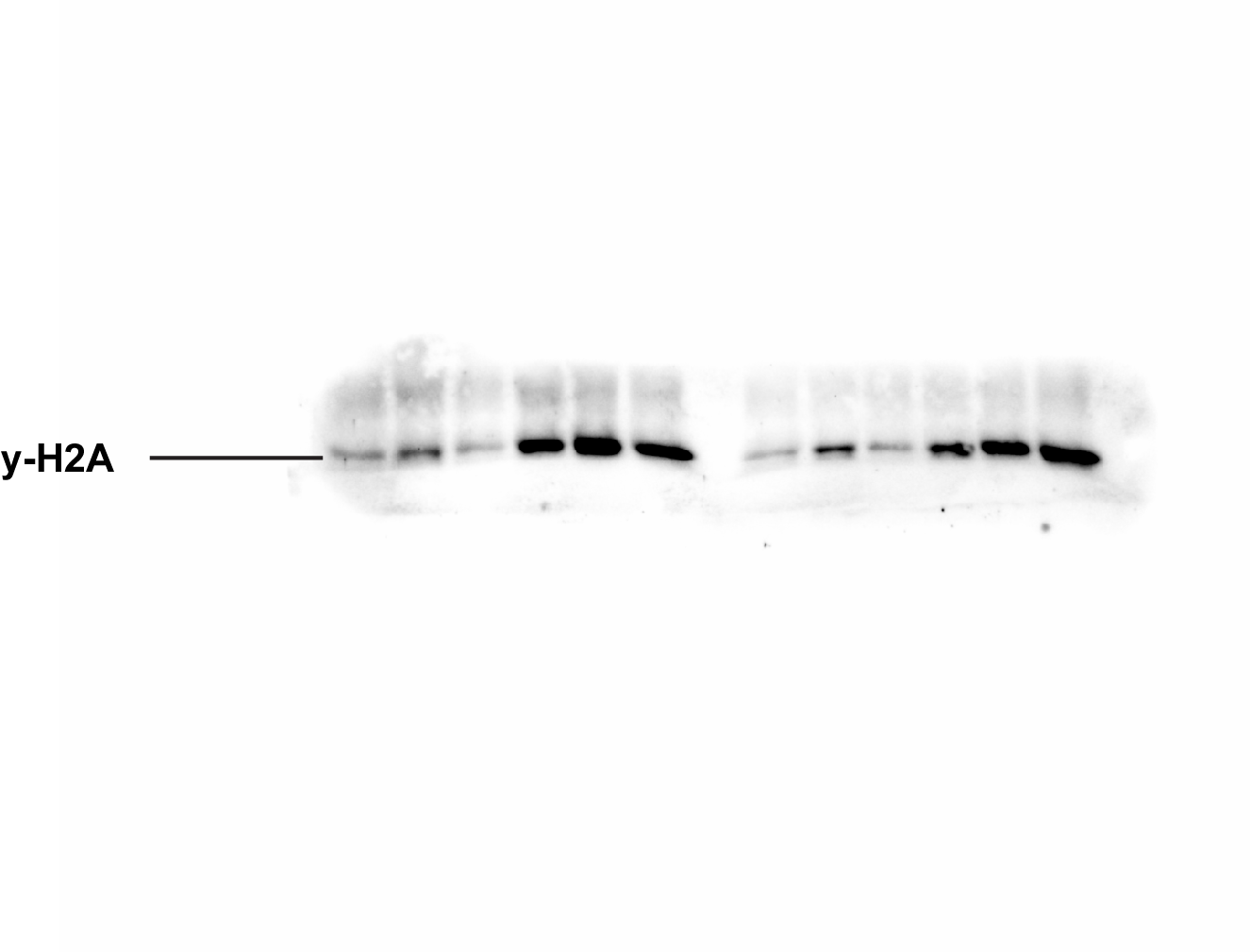

Supplement: Figure 6—figure supplement 1—source data 2. [file elife-104431-fig6-figsupp1-data2.zip › Figure 6- figure supplement 1 /Figure 6-source data-Supp 6F H2A.tif]

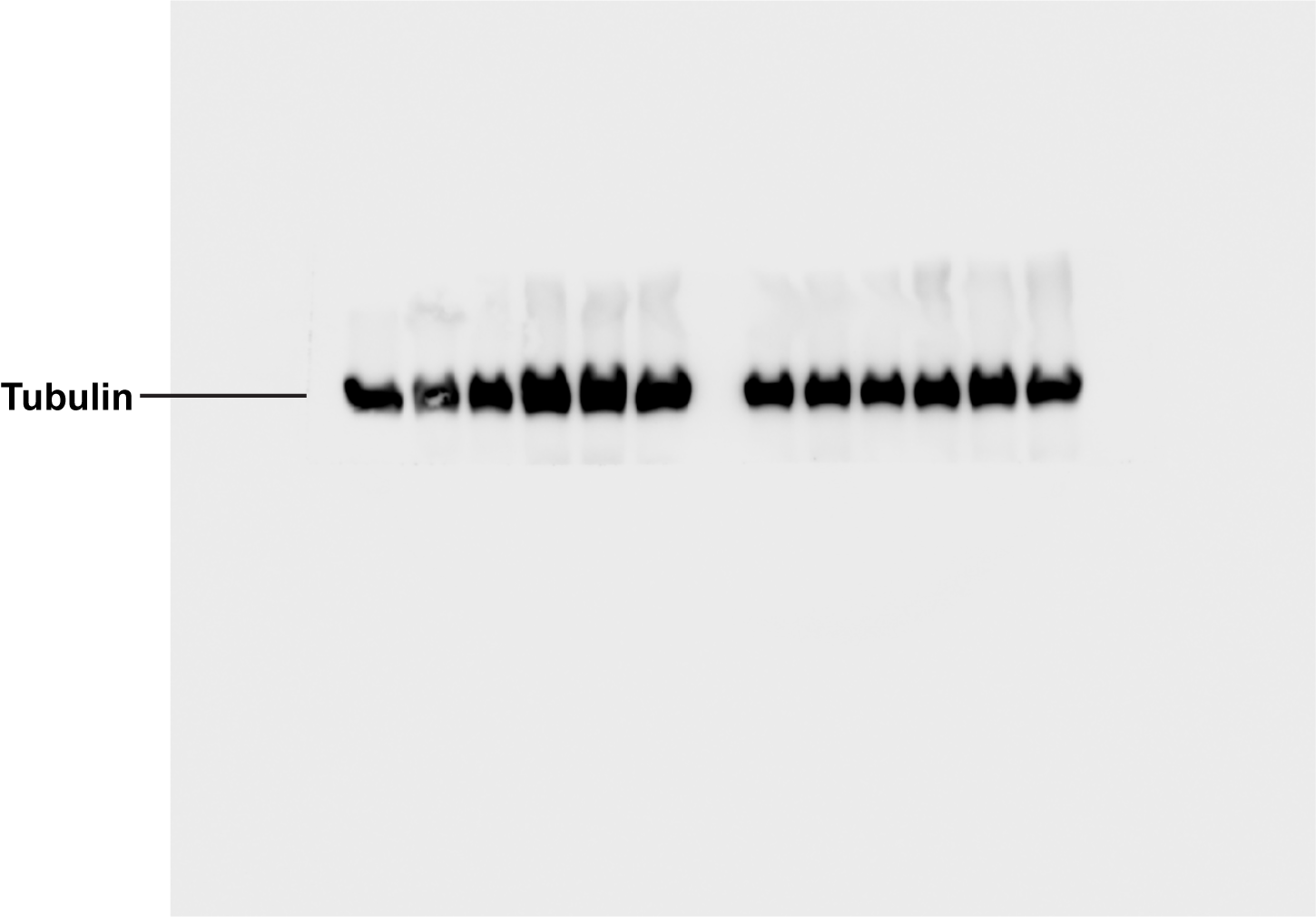

Supplement: Figure 6—figure supplement 1—source data 2. [file elife-104431-fig6-figsupp1-data2.zip › Figure 6- figure supplement 1 /Figure 6-source data-Supp 6F Tub.tif]

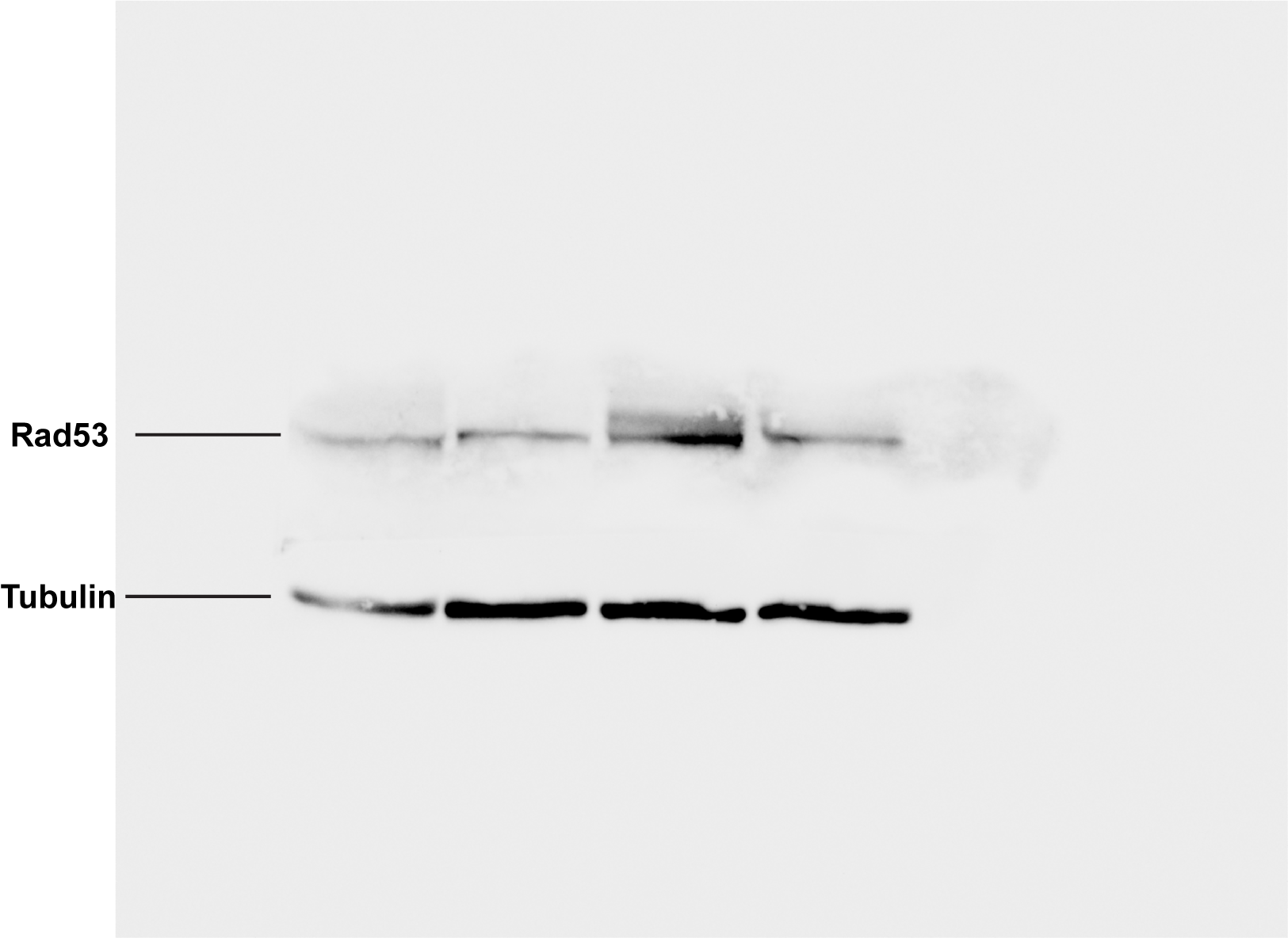

Supplement: Figure 6—figure supplement 1—source data 2. [file elife-104431-fig6-figsupp1-data2.zip › Figure 6- figure supplement 1 /Figure 6-source data-Supp 6B.tif]

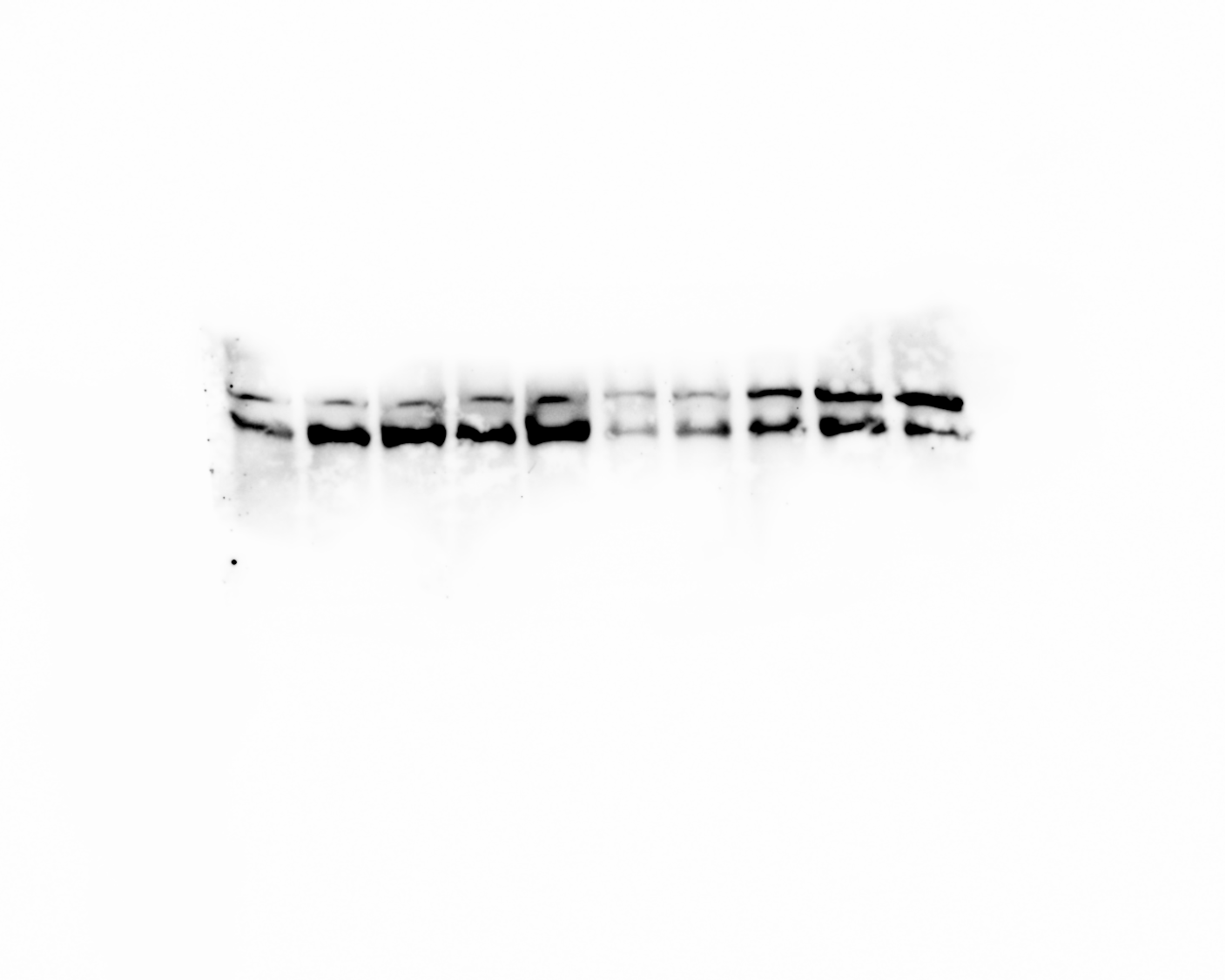

Supplement: Figure 7—source data 1. [file elife-104431-fig7-data1.zip › Figure 7/Figure 7-source data-7D_Scm3.tif]

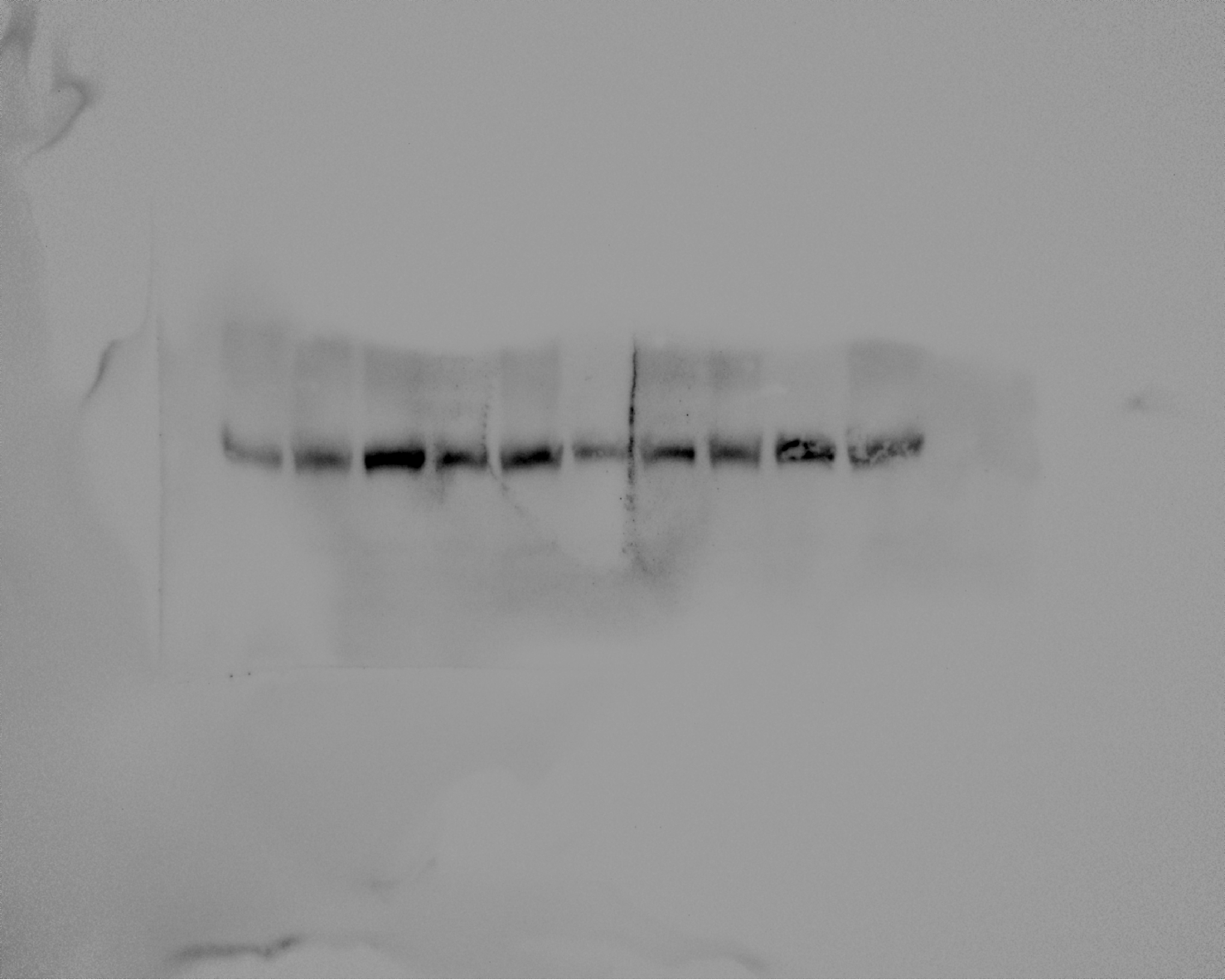

Supplement: Figure 7—source data 1. [file elife-104431-fig7-data1.zip › Figure 7/Figure 7-souce data- 7D.tif]

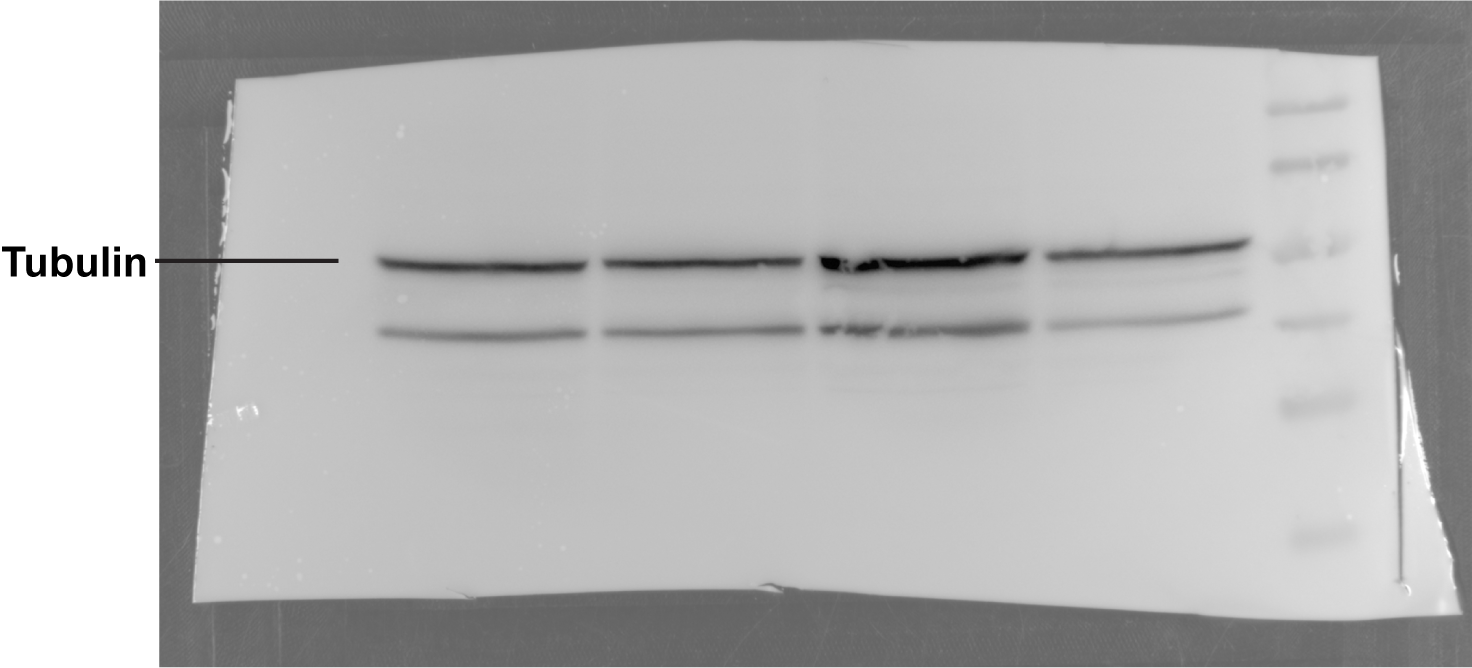

Supplement: Figure 7—source data 2. [file elife-104431-fig7-data2.zip › Figure 7/Figure 7-source data- 7A Tub.tif]

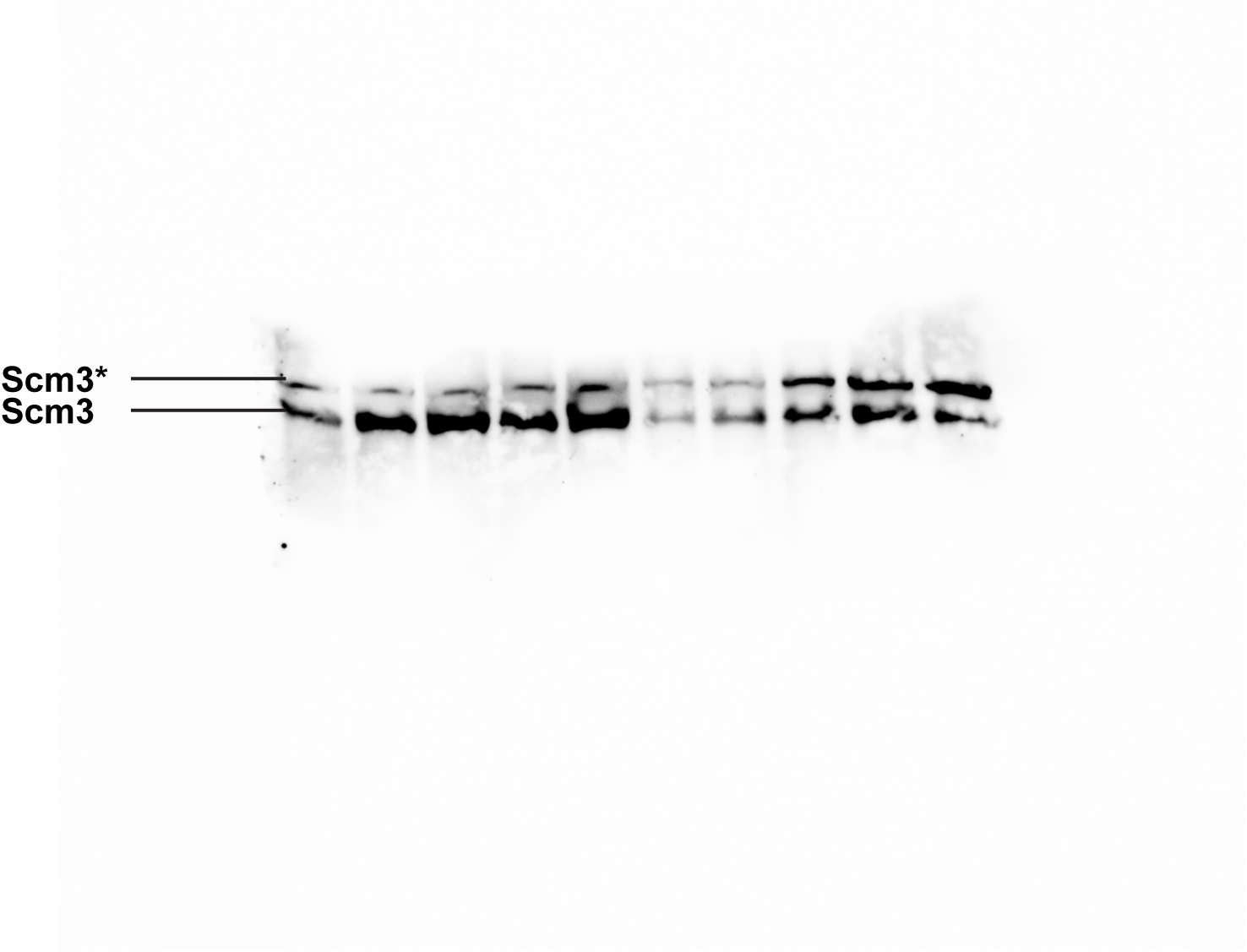

Supplement: Figure 7—source data 2. [file elife-104431-fig7-data2.zip › Figure 7/Figure 7-source data-7D_Scm3.tif]

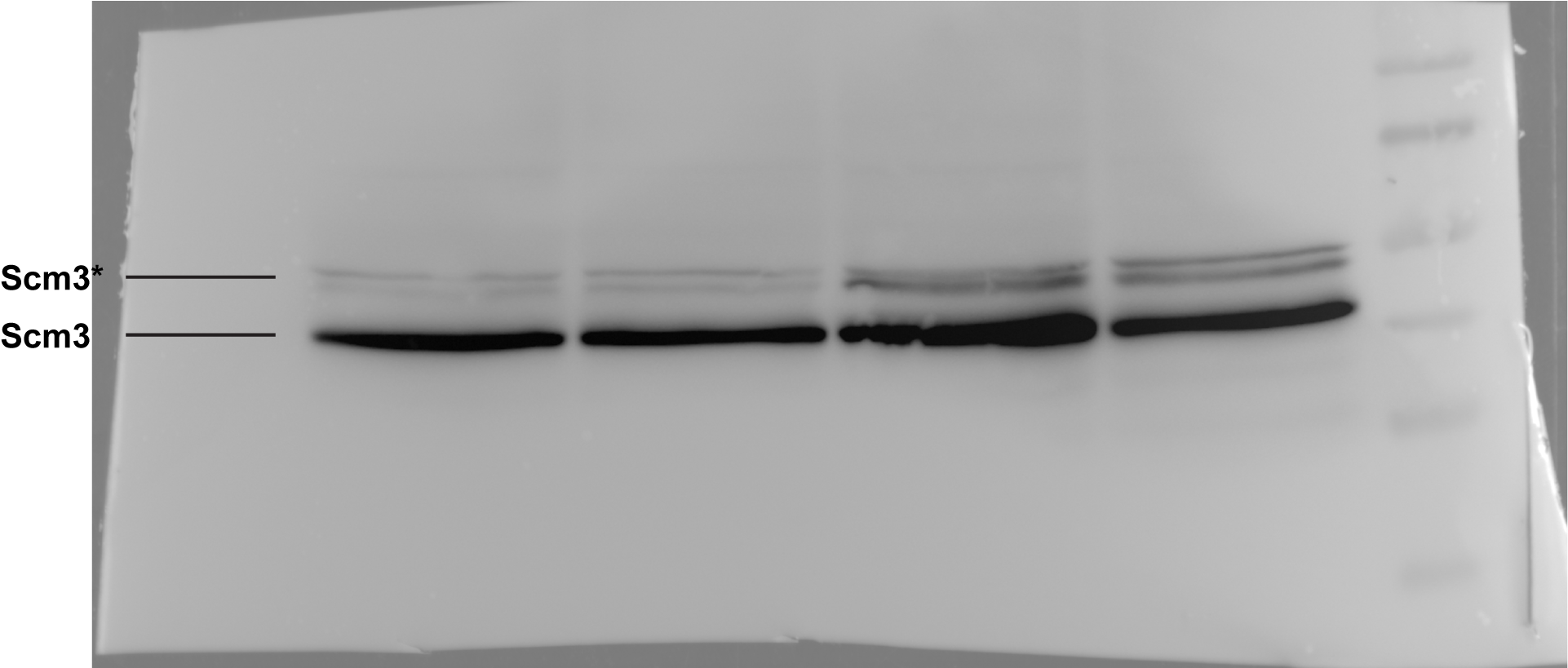

Supplement: Figure 7—source data 2. [file elife-104431-fig7-data2.zip › Figure 7/Figure 7- source data- 7A Scm3.tif]

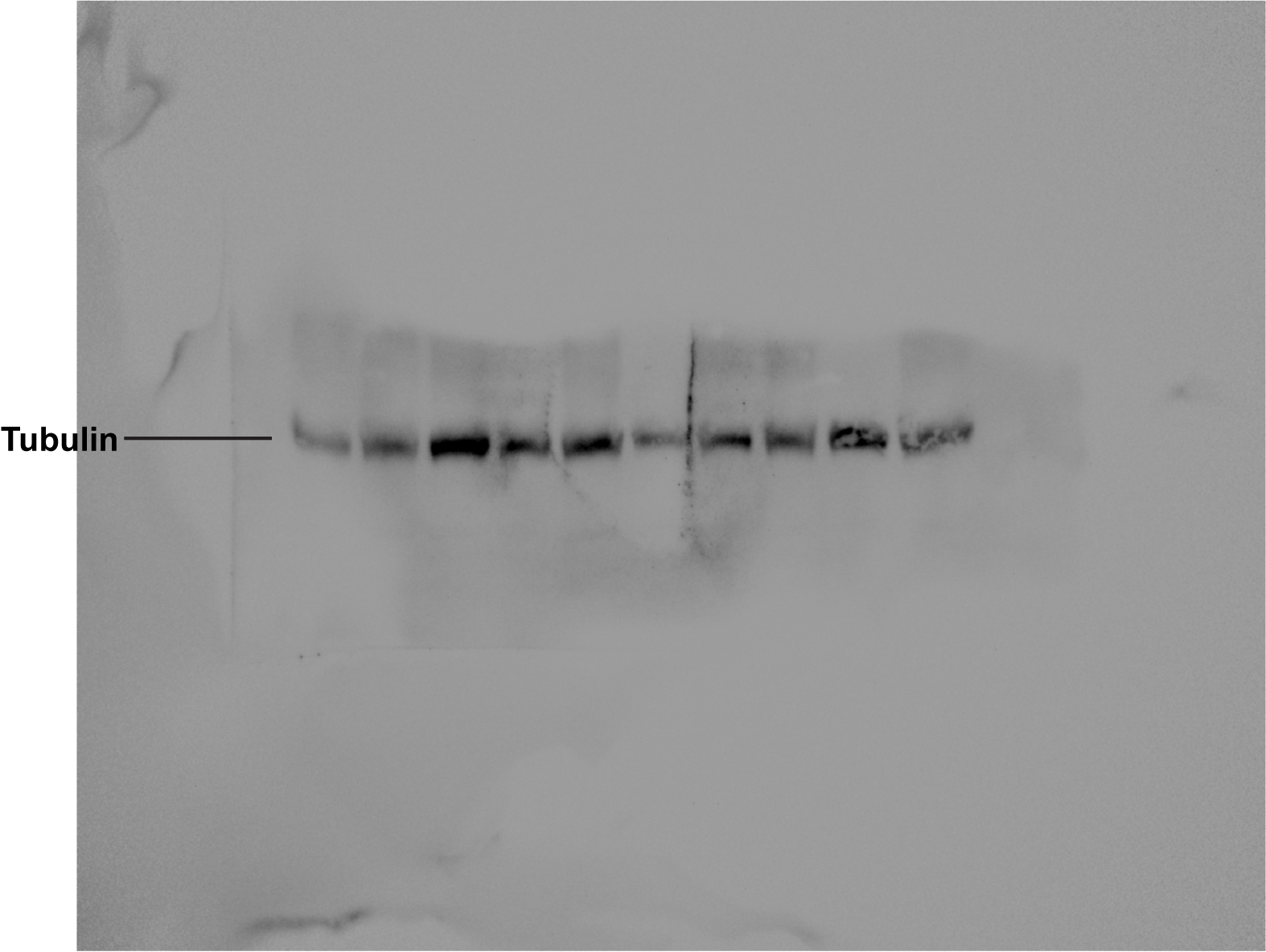

Supplement: Figure 7—source data 2. [file elife-104431-fig7-data2.zip › Figure 7/Figure 7-souce data- 7D.tif]

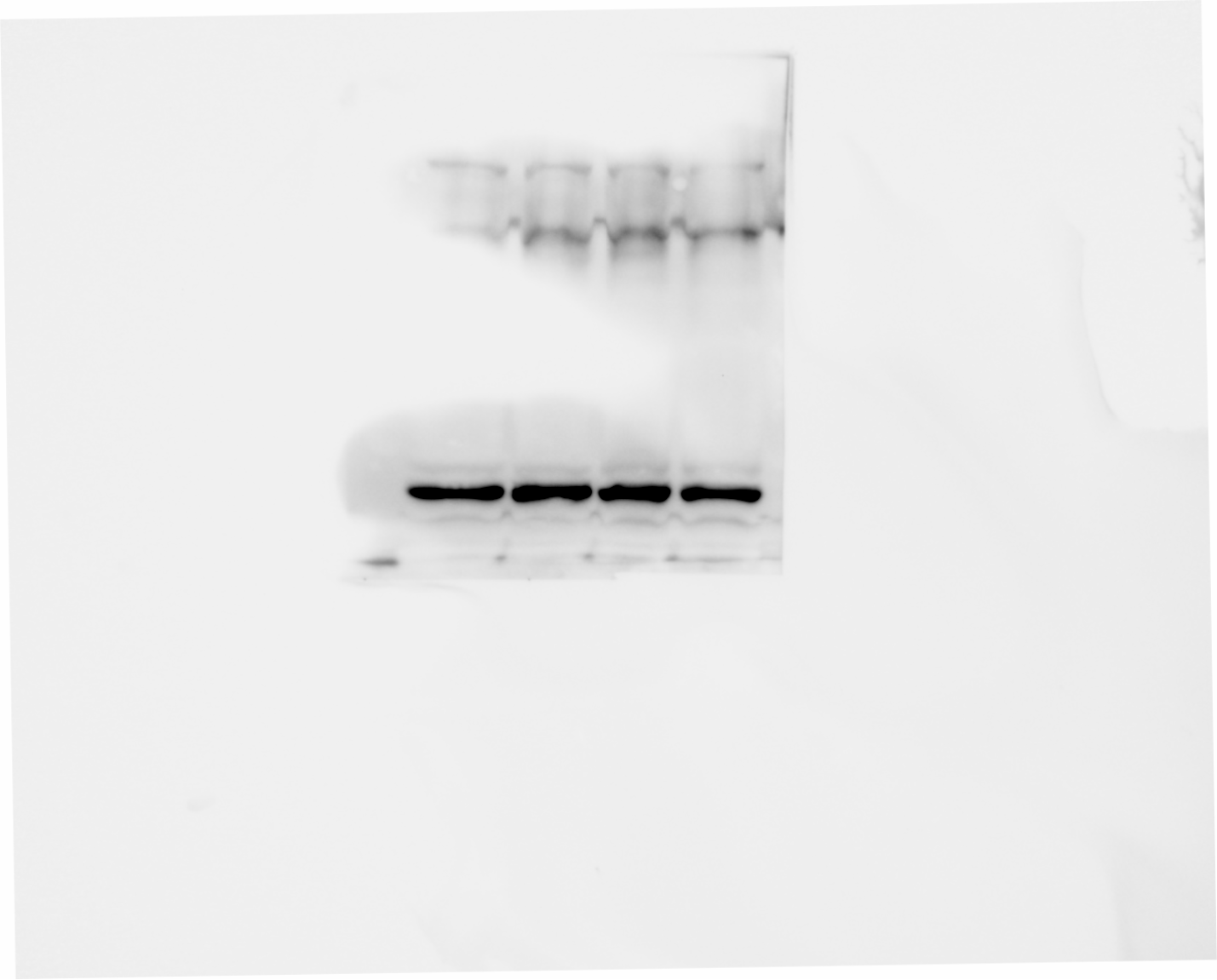

Supplement: Figure 7—figure supplement 1—source data 1. [file elife-104431-fig7-figsupp1-data1.zip › Figure 7- figure supplement 1 /Figure 7-source data-Supp 7D Untreated.tif]

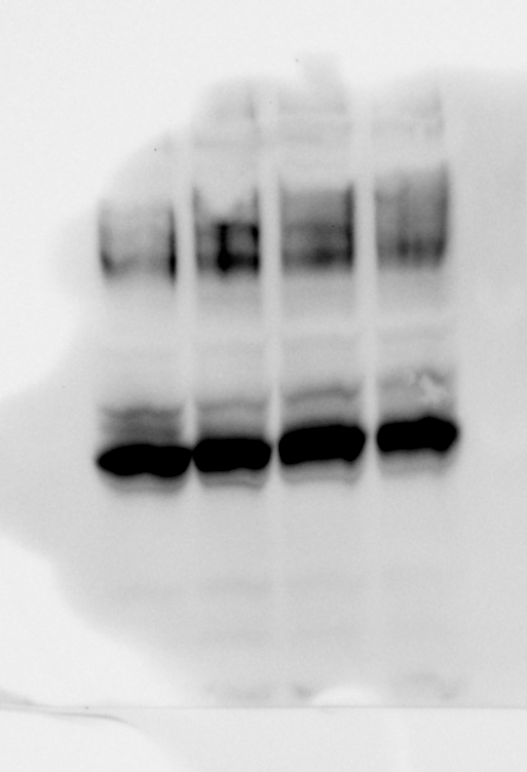

Supplement: Figure 7—figure supplement 1—source data 1. [file elife-104431-fig7-figsupp1-data1.zip › Figure 7- figure supplement 1 /Figure 7-source data-Supp 7D Treated.tif]

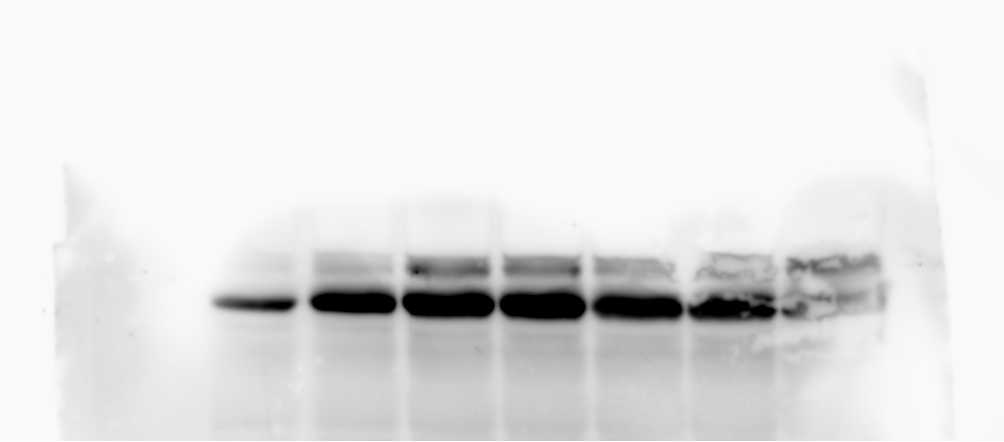

Supplement: Figure 7—figure supplement 1—source data 1. [file elife-104431-fig7-figsupp1-data1.zip › Figure 7- figure supplement 1 /Figure 7-source data-Supp 7A Scm3.tif]

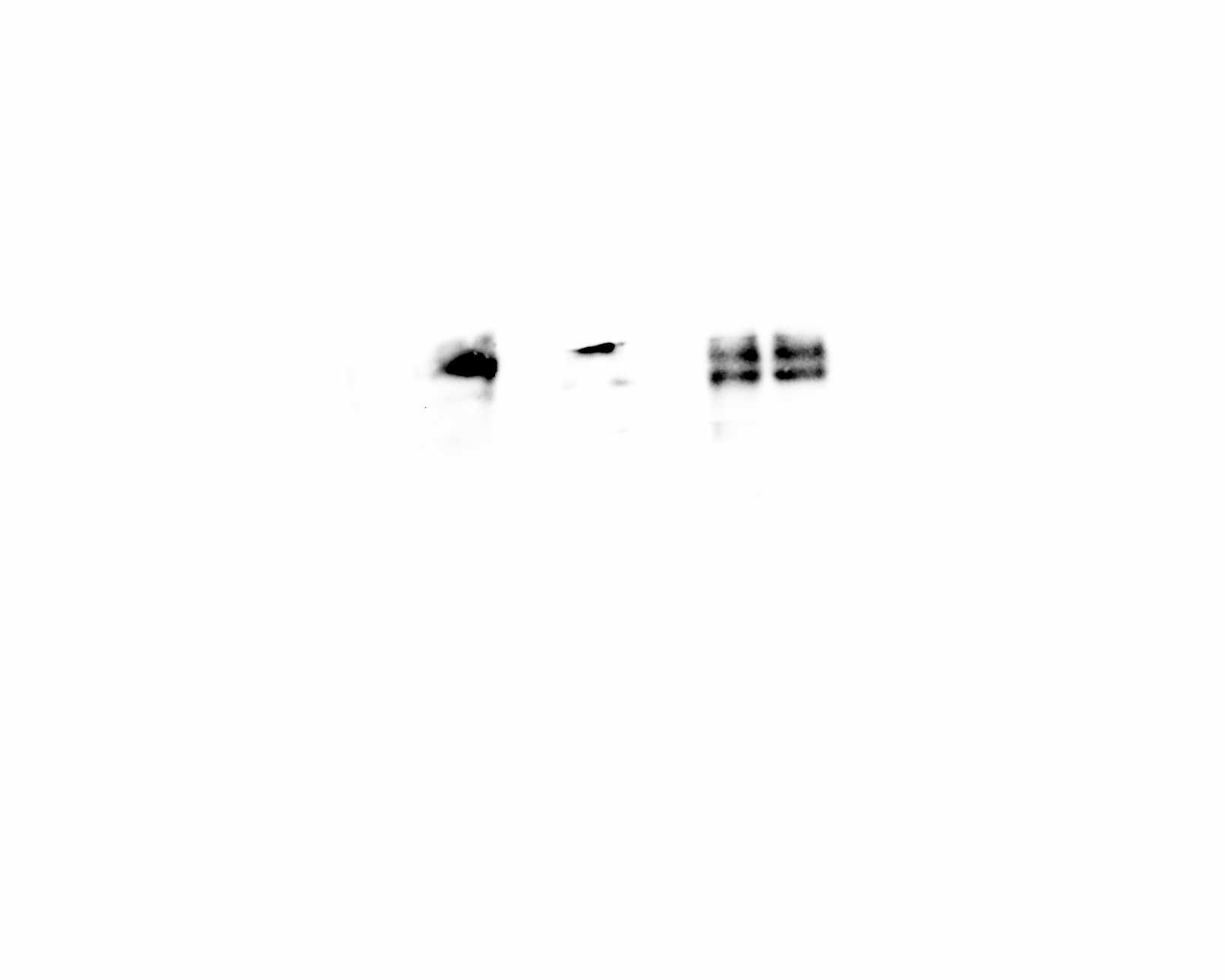

Supplement: Figure 7—figure supplement 1—source data 1. [file elife-104431-fig7-figsupp1-data1.zip › Figure 7- figure supplement 1 /tel1-2.tif]

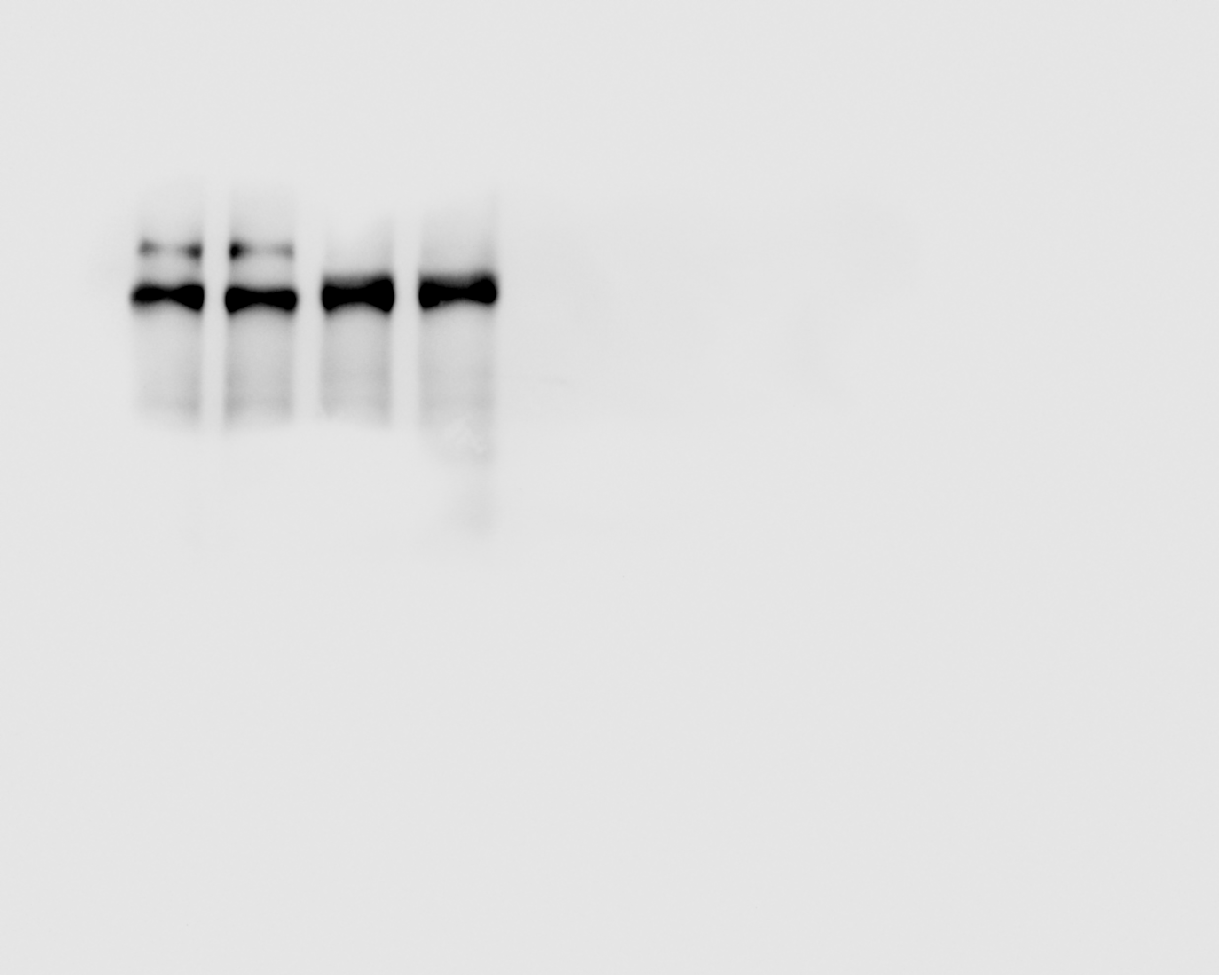

Supplement: Figure 7—figure supplement 1—source data 1. [file elife-104431-fig7-figsupp1-data1.zip › Figure 7- figure supplement 1 /Figure 7-source data-Supp 7F Phospha.tif]

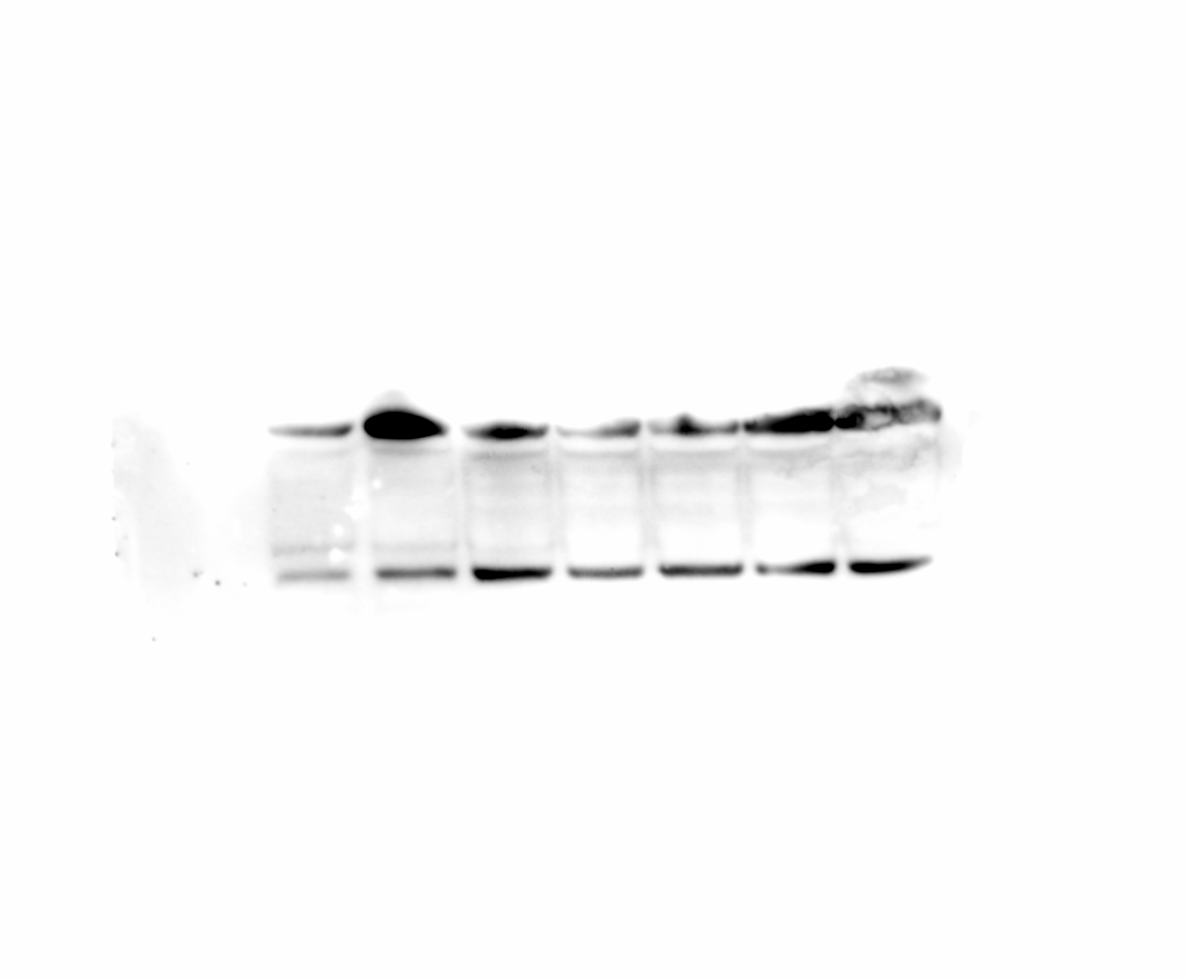

Supplement: Figure 7—figure supplement 1—source data 1. [file elife-104431-fig7-figsupp1-data1.zip › Figure 7- figure supplement 1 /Figure 7-source data-Supp 7A Tub2.tif]

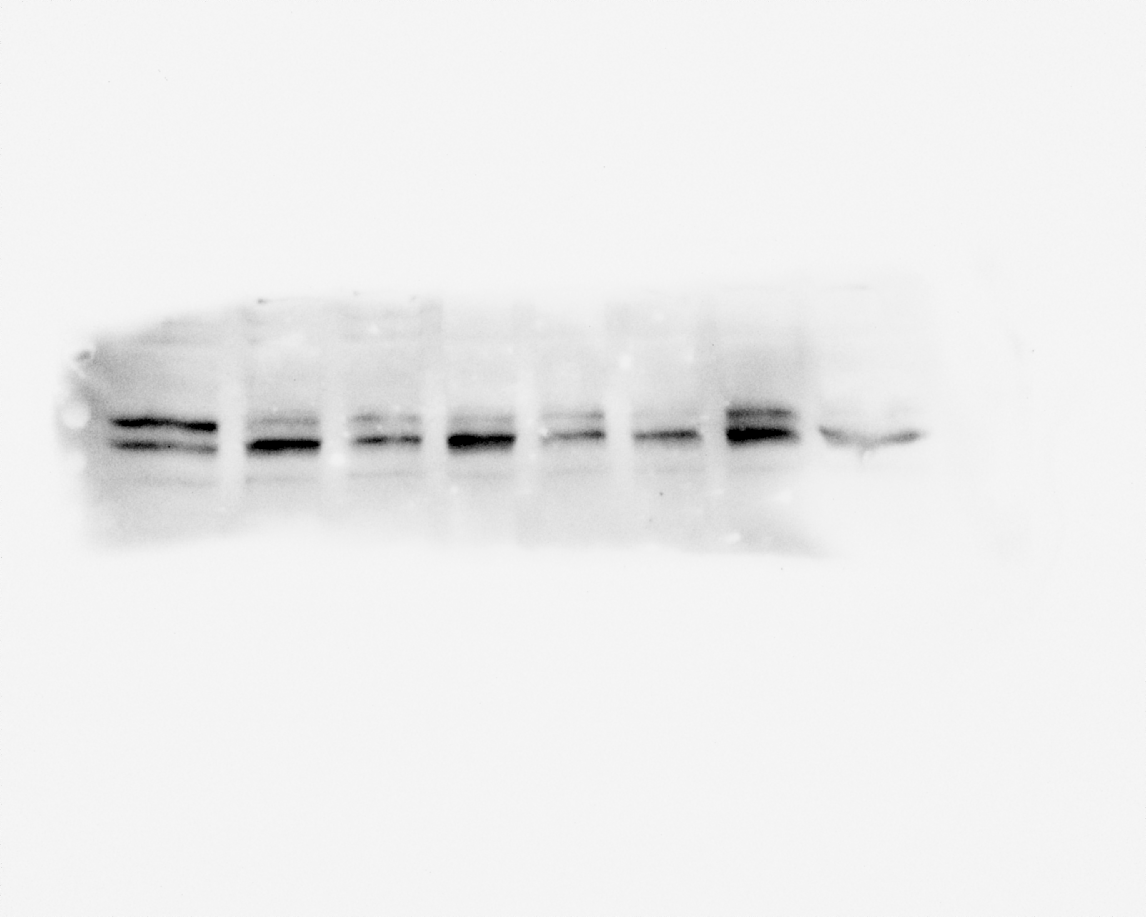

Supplement: Figure 7—figure supplement 1—source data 1. [file elife-104431-fig7-figsupp1-data1.zip › Figure 7- figure supplement 1 /Figure 7-source data-Supp 7G Mec1.tif]

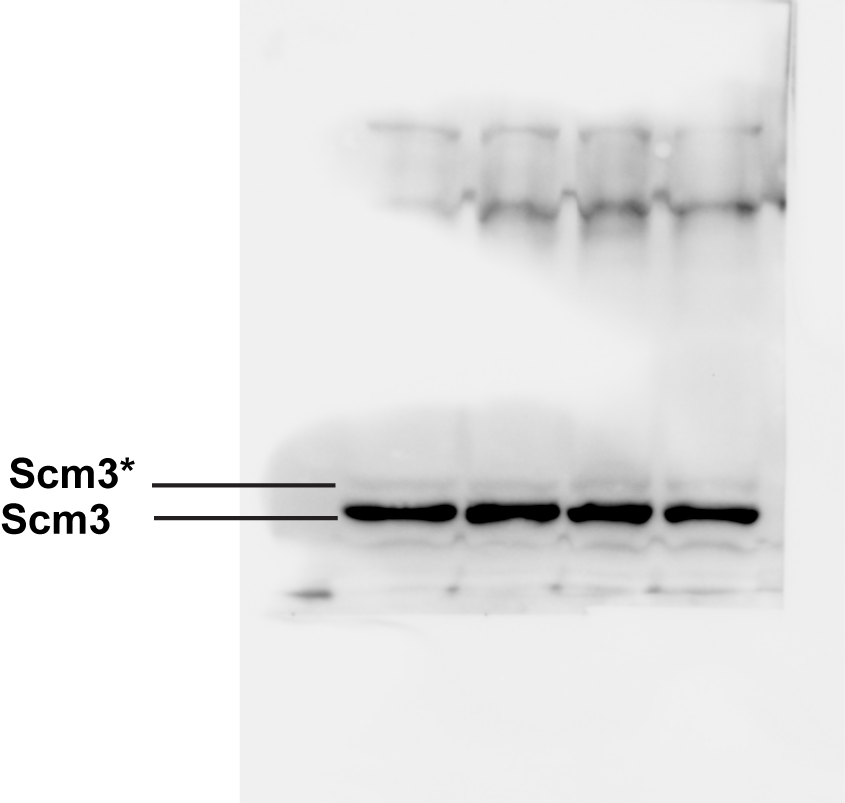

Supplement: Figure 7—figure supplement 1—source data 2. [file elife-104431-fig7-figsupp1-data2.zip › Figure 7- figure supplement 1 /Figure 7-source data-Supp 7D Untreated.tif]

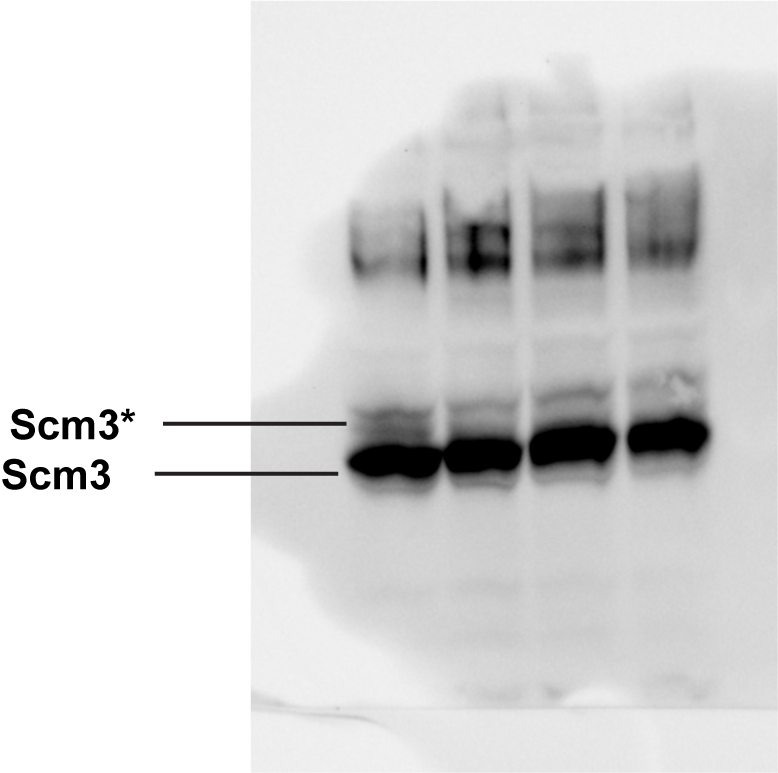

Supplement: Figure 7—figure supplement 1—source data 2. [file elife-104431-fig7-figsupp1-data2.zip › Figure 7- figure supplement 1 /Figure 7-source data-Supp 7D Treated.tif]

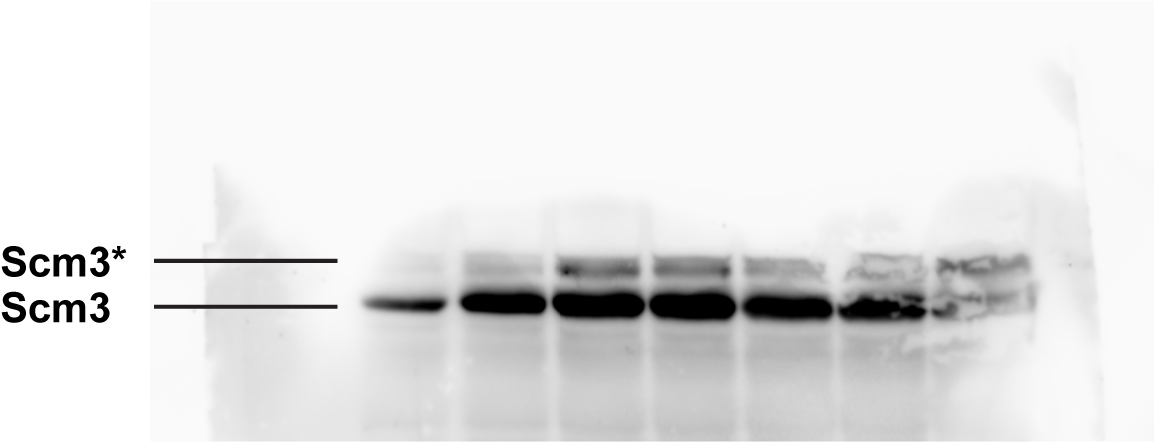

Supplement: Figure 7—figure supplement 1—source data 2. [file elife-104431-fig7-figsupp1-data2.zip › Figure 7- figure supplement 1 /Figure 7-source data-Supp 7A Scm3.tif]

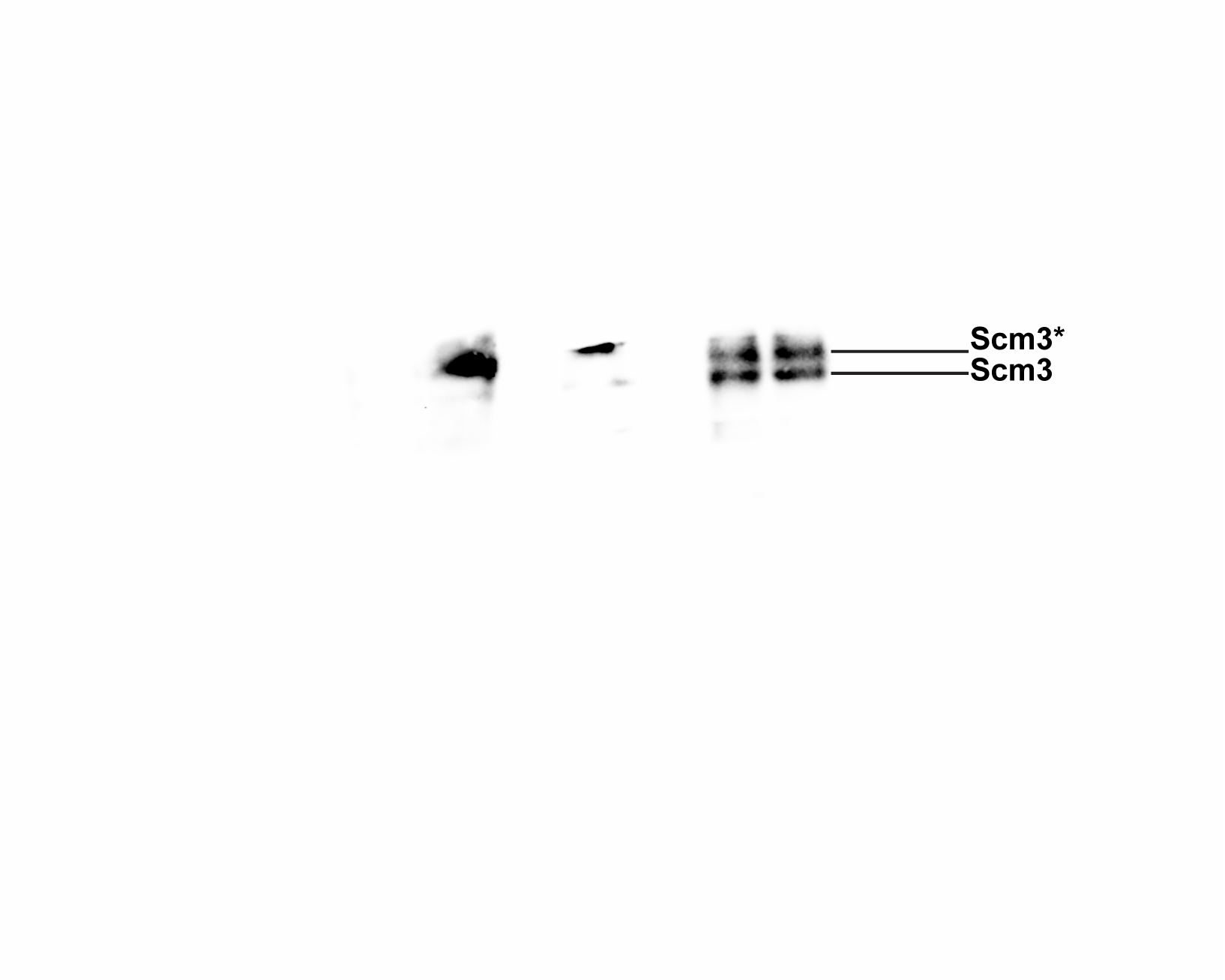

Supplement: Figure 7—figure supplement 1—source data 2. [file elife-104431-fig7-figsupp1-data2.zip › Figure 7- figure supplement 1 /tel1-2.tif]

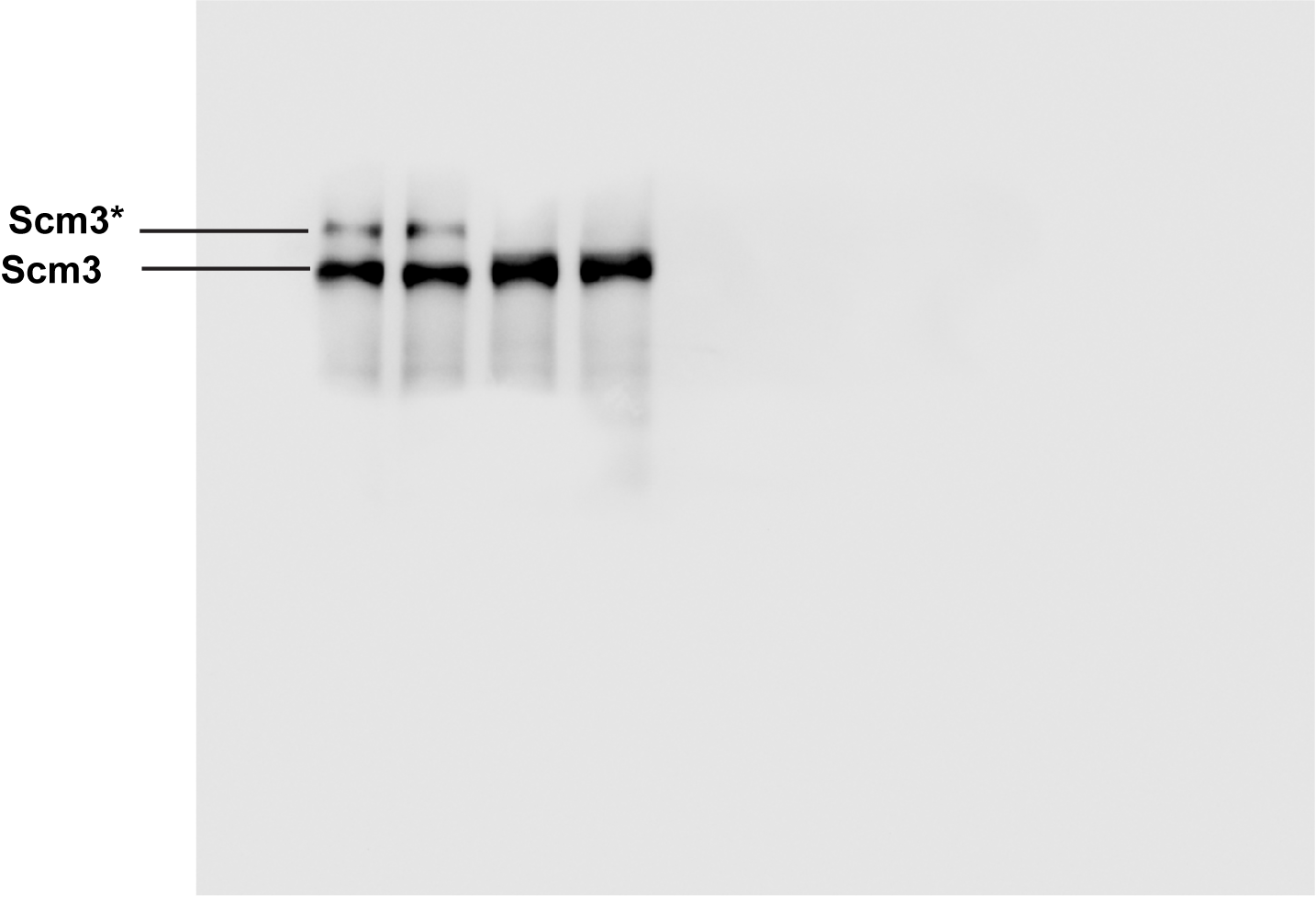

Supplement: Figure 7—figure supplement 1—source data 2. [file elife-104431-fig7-figsupp1-data2.zip › Figure 7- figure supplement 1 /Figure 7-source data-Supp 7F Phospha.tif]

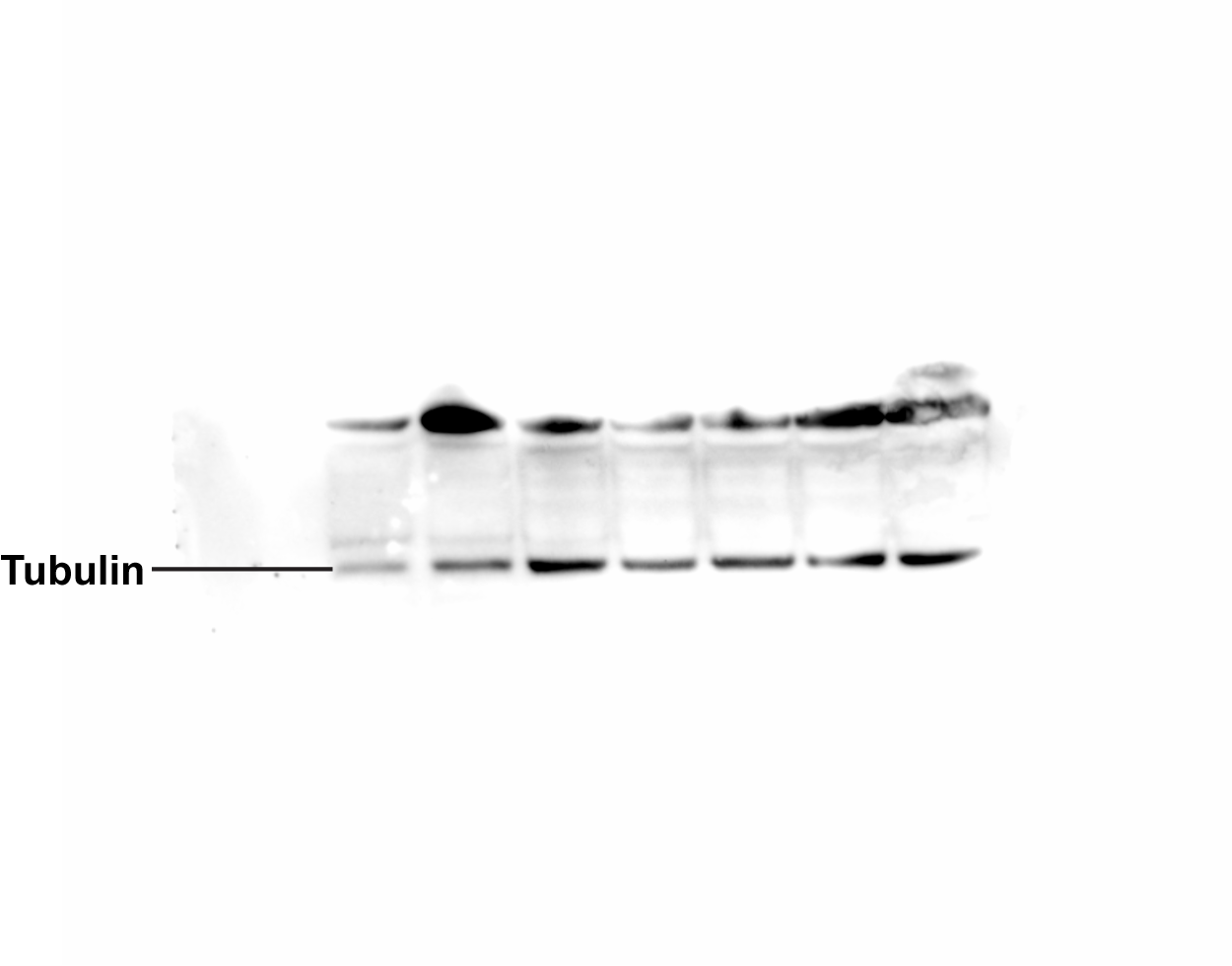

Supplement: Figure 7—figure supplement 1—source data 2. [file elife-104431-fig7-figsupp1-data2.zip › Figure 7- figure supplement 1 /Figure 7-source data-Supp 7A Tub2.tif]

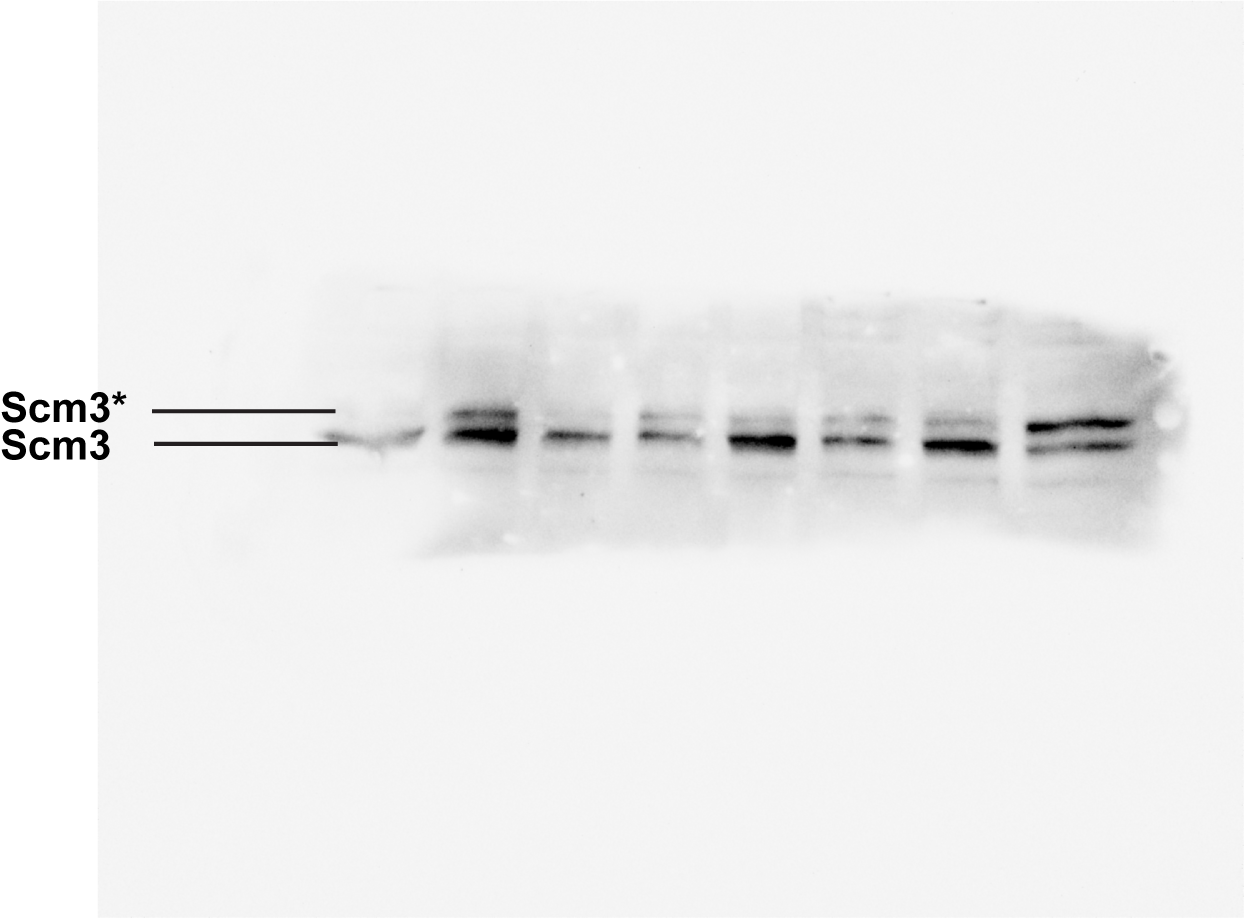

Supplement: Figure 7—figure supplement 1—source data 2. [file elife-104431-fig7-figsupp1-data2.zip › Figure 7- figure supplement 1 /Figure 7-source data-Supp 7G Mec1.tif]
