# Supplementary material for: Evidence of centromeric histone 3 chaperone involved in DNA damage repair pathway in budding yeast
Source: eLife. 2025 Dec 10;14:e104431. doi: 10.7554/eLife.104431 (PMC12695027; doi:10.7554/eLife.104431)
Supplement: Supplementary file 1. — (A) List of strains used in this study. (B) List of primers used in ChIP-qPCR. [file elife-104431-supp1.docx]

**Supplementary material**

**Supplementary file 1A: List of strains used in this study:**

| **S.no.** | Name | **Genotype** | Source |
| --- | --- | --- | --- |
| **1.** | CRY1 | *MATa, ura3-1 leu2,3-112 his3-1 trp1-1 ade2-1 can1-100* | (Rothstein, 1983) |
| **2.** | SGY10016 | *MATa, ura3-1 leu2,3-112 his3-1 trp1-1 ade2-1 can1-100 rad52∆::KanMx* | This study |
| **3.** | SGY10035 | *MATa, ura3-1 leu2,3-112 his3-1 trp1-1 ade2-1 can1-100 SCM3-AID::KanMx, pADH-Os-TIR1::URA3* | This study |
| **4.** | SGY10037 | *MATa, ura3-1 leu2,3-112 his3-1 trp1-1 ade2-1 can1-100 SCM3-AID::KanMx,pADH-Os-TIR1::URA3, rad52∆::HPHMX* | This study |
| **5.** | SGY10157 | *MATa, ura3-1 leu2,3-112 his3-1 trp1-1 ade2-1 can1-100 ctf19∆::HPHMX* | This study |
| **6.** | SGY71 | *MATa, ho::Lys2, lys2, ura3, leu2::hisG, his3::hisG, trp1::hisG, MIF2-AID::KanMx, pADH-Os-TIR1::URA* | (Mehta et al., 2014) |
| **7.** | SGY79 | *MATa, ho::Lys2, lys2, ura3, leu2::hisG, his3::hisG, trp1::hisG, NDC10-AID::KanMx, pADH-Os-TIR1::URA* | (Mehta et al., 2014) |
| **8.** | SGY10044 | *MATa, ura3-1 leu2,3-112 his3-1 trp1-1 ade2-1 can1-100 SCM3-AID::KanMx, pADH-Os-TIR1::URA, RAD52-GFP::TRP1* | This study |
| **9.** | SGY10061 | *MATa, ura3-1 leu2,3-112 his3-1 trp1-1 ade2-1 can1-100 SCM3-AID::KanMx, pADH-Os-TIR1::URA, RAD52-GFP::TRP1, bar1∆::LEU2* | This study |
| **10.** | SGY10065 | *MATa, ura3-1 leu2,3-112 his3-1 trp1-1 ade2-1 can1-100 NDC10-6HA::HIS3, SCM3-13MYC::KanMx* | This study |
| **11.** | SGY10063 | *MATa, ura3-1 leu2,3-112 his3-1 trp1-1 ade2-1 can1-100 RAD52-6HA::HIS3, SCM3-13MYC::KanMx* | This study |
| **12.** | NA14 | *MATa-inc ura3-HOcs lys2::ura3-HOcs-inc ade2-1 ade3:: GALHO leu2-3112 his3-11,15 trp1-1 can1-100* | (Agmon et al., 2009; Fangaria et al., 2022) |
| **13.** | SGY10179 | *NA14, ura3-1 leu2,3-112 his3-1 trp1-1 ade2-1 can1-100 SCM3-6HA::HPHMX* | This study |
| **14.** | SGY10032 | *MATa, ura3-1 leu2,3-112 his3-1 trp1-1 ade2-1 can1-100 CDC20-AID::KanMx, pADH-Os-TIR1::URA3* | This study |
| **15.** | SGY10038 | *MATa, ura3-1 leu2,3-112 his3-1 trp1-1 ade2-1 can1-100 SCM3-AID::KanMx, pADH-Os-TIR1::URA3, mad2∆::HPHMX* | This study |
| **16.** | SGY10019 | *MATa, ura3-1 leu2,3-112 his3-1 trp1-1 ade2-1 can1-100 SCM3-6HA::HIS3* | This study |
| **17.** | SGY10059 | *MATa, ura3-1 leu2,3-112 his3-1 trp1-1 ade2-1 can1-100 SCM3-6HA::HIS3 bar1∆::LEU2* | This study |
| **18.** | SGY10088 | *MATa, ura3-1 leu2,3-112 his3-1 trp1-1 ade2-1 can1-100 SCM3-6HA::HIS3 sml1∆::LEU2* | This study |
| **19.** | SGY10089 | *MATa, ura3-1 leu2,3-112 his3-1 trp1-1 ade2-1 can1-100 SCM3-6HA::HIS3 sml1∆::LEU2 mec1∆::KanMx* | This study |
| **20.** | SGY10119 | *MATa, ura3-1 leu2,3-112 his3-1 trp1-1 ade2-1 can1-100 Δbar1 scm3Δ::TRP1 pRS423-SCM3::HIS3* | This study |
| **21.** | SGY10120 | *MATa ura3-1 leu2,3-112 his3-1 trp1-1 ade2-1 can1-100 Δbar1 scm3Δ::TRP1 pRS423-scm3-Δ25C::HIS3* | This study |
| **22.** | SGY10121 | *MATa ura3-1 leu2,3-112 his3-1 trp1-1 ade2-1 can1-100 Δbar1 scm3Δ::TRP1 pRS423-scm3-ΔNLS::HIS3* | This study |
| **23.** | SGY10212 | *MATa, ura3-1 leu2,3-112 his3-1 trp1-1 ade2-1 can1-100 RAD52-GFP::TRP1* | This study |
| **24.** | SGY10211 | *MATa, ura3-1 leu2,3-112 his3-1 trp1-1 ade2-1 can1-100 HSF1-13MYC::KanMx* | This study |
| **25.** | SGY10209 | *MATa, ura3-1 leu2,3-112 his3-1 trp1-1 ade2-1 can1-100 SCM3-AID::KanMx, pADH-Os-TIR1::URA3, bar1∆::LEU2, mad2∆::HPHMX, PDS1-13MYC::TRP1* | This study |
| **26.** | SGY10210 | *MATa, ura3-1 leu2,3-112 his3-1 trp1-1 ade2-1 can1-100 tel1∆::LEU2 sml1∆::KanMx SCM3-6HA::HIS3* | This study |
| **27.** | SGY10211 | *MATa, ura3-1 leu2,3-112 his3-1 trp1-1 ade2-1 can1-100 tel1∆::LEU2 sml1∆::KanMx mec1∆::HPHMX SCM3-6HA::HIS3* | This study |
| **28.** | SGY10212 | *NA14, ura3-1 leu2,3-112 his3-1 trp1-1 ade2-1 can1-100 SCM3-6HA-AID::HPHMX pADH-Os-TIR1::URA3* | This study |
| **29.** | SGY10213 | *MATa, ura3-1 leu2,3-112 his3-1 trp1-1 ade2-1 can1-100 SCM3-13MYC::HPHMX tel1∆::LEU2 sml1∆::KanMx mec1∆::TRP1* | This study |

**Supplementary file 1B: List of primers used in ChIP-qPCR:**

| **Name** | **Description** | **Primer sequence (5′-3′)** | **Source** |
| --- | --- | --- | --- |
| *CEN3* | Forward | GATCAGCGCCAAACAATATGG | (Mehta et al., 2014) |
|  | Reverse | AACTTCCACCAGTAAACGTTT |  |
| *CEN4* | Forward | GCTTGCAAAAGGTCACATGC | (Mehta et al., 2014) |
|  | Reverse | GAGCAGGTTTTATGTTTCGG |  |
| *TUB2* | Forward | CTTGTAGACAGCGTCATGG | (Mehta et al., 2014) |
|  | Reverse | CAGATGTCATAAAGTGCTTCG |  |
| DSB | Forward (OSB289) | GTTAGTTGAAGCATTAGGTCC | (Fangaria et al., 2022) |
|  | Reverse (kanB1) | TGTACGGGCGACAGTCACAT |  |
| Near DSB | Forward | ATGTCGAAAGCTACATATAAG | (Fangaria et al., 2022) |
|  | Reverse | AATGCTTCAACTAACTCCAG |  |
| -1 kb | Forward | GGAGAATCCATACAAGAAATCG | (Fangaria et al., 2022) |
|  | Reverse | CATCTCATTAGTTGGAATTTCG |  |
| -2 kb | Forward | TTTGGTAGATCATTTAAGGGTC | (Fangaria et al., 2022) |
|  | Reverse | CAGGAGATGGCTTAGGCAAG |  |
| -3 kb | Forward | TTTCATTGCTTCCGACTCCG | (Fangaria et al., 2022) |
|  | Reverse | TACGCAGACGAGAAGGCTTC |  |

**Supplementary References:**

- Agmon, N., Pur, S., Liefshitz, B., & Kupiec, M. (2009). Analysis of repair mechanism choice during homologous recombination. *Nucleic Acids Research*, *37*(15), 5081–5092. https://doi.org/10.1093/NAR/GKP495
- Fangaria, N., Rani, K., Singh, P., Dey, S., Kumar, K. A., & Bhattacharyya, S. (2022). DNA damage-induced nuclear import of HSP90α is promoted by Aha1. *Molecular Biology of the Cell*, *33*(14). https://doi.org/10.1091/MBC.E21-11-0554
- Mehta, G. D., Agarwal, M., & Ghosh, S. K. (2014b). Functional characterisation of kinetochore protein, Ctf19 in meiosis I: an implication of differential impact of Ctf19 on the assembly of mitotic and meiotic kinetochores in Saccharomyces cerevisiae. *Molecular Microbiology*, *91*(6), 1179–1199. https://doi.org/10.1111/mmi.12527
- Rothstein, R. J. (1983). *One-step gene disruption in yeast* (pp. 202–211). https://doi.org/10.1016/0076-6879(83)01015-0
